# Compartmentalization of Mammalian Pantothenate Kinases

**DOI:** 10.1371/journal.pone.0049509

**Published:** 2012-11-13

**Authors:** Adolfo Alfonso-Pecchio, Matthew Garcia, Roberta Leonardi, Suzanne Jackowski

**Affiliations:** Department of Infectious Diseases, St. Jude Children’s Research Hospital, Memphis, Tennessee, United States of America; Medical University of South Carolina, United States of America

## Abstract

The pantothenate kinases (PanK) catalyze the first and the rate-limiting step in coenzyme A (CoA) biosynthesis and regulate the amount of CoA in tissues by differential isoform expression and allosteric interaction with metabolic ligands. The four human and mouse PanK proteins share a homologous carboxy-terminal catalytic domain, but differ in their amino-termini. These unique termini direct the isoforms to different subcellular compartments. PanK1α isoforms were exclusively nuclear, with preferential association with the granular component of the nucleolus during interphase. PanK1α also associated with the perichromosomal region in condensing chromosomes during mitosis. The PanK1β and PanK3 isoforms were cytosolic, with a portion of PanK1β associated with clathrin-associated vesicles and recycling endosomes. Human PanK2, known to associate with mitochondria, was specifically localized to the intermembrane space. Human PanK2 was also detected in the nucleus, and functional nuclear localization and export signals were identified and experimentally confirmed. Nuclear PanK2 trafficked from the nucleus to the mitochondria, but not in the other direction, and was absent from the nucleus during G2 phase of the cell cycle. The localization of human PanK2 in these two compartments was in sharp contrast to mouse PanK2, which was exclusively cytosolic. These data demonstrate that PanK isoforms are differentially compartmentalized allowing them to sense CoA homeostasis in different cellular compartments and enable interaction with regulatory ligands produced in these same locations.

## Introduction

Coenzyme A (CoA) is an essential cofactor involved in lipid and energy metabolism that carries organic acid substrates and supports a multitude of oxidative and synthetic metabolic reactions, including those involved in the citric acid cycle, sterol, bile acid, fatty acid and lipid synthesis, fatty acid oxidation and lipolysis. CoA is derived from vitamin B5 (pantothenate), cysteine and ATP. Pantothenate kinases (PanKs) catalyze the first regulatory step in CoA synthesis, and the remaining biosynthetic steps are catalyzed by cytosolic enzymes found either soluble or associated with the cytosolic aspect of the outer mitochondrial membrane [1]. There is one PanK gene in most bacteria, fungi, and flies, whereas three genes express four catalytically active isoforms in mammals: PanK1α, PanK1β, PanK2 and PanK3 [1]. A putative PanK4 does not appear to be catalytically active [2]. The α and β isoforms of PanK1 are encoded by different transcripts that arise from alternate initiation exons within the *Pank1* gene [3]. The expression of PanK1 and PanK2 isoforms differs among tissues, but PanK3 is found in all cell types examined thus far.

The physiological significance of PanK function is best evidenced by the fact that PanK1β is most highly expressed in liver and *Pank1* knockout mice are unable to fully transition to fasting metabolism due to impaired hepatic fatty acid oxidation and reduced gluconeogenesis [4]. In addition, human PanK2 is highly expressed in the brain [5] and mutations in the human *PANK2* gene result in a progressive neurodegenerative disease, called PKAN (Pantothenate Kinase Associated Neurodegeneration) [6]. PKAN is an autosomal recessive disorder associated with iron accumulation in the brain and characterized by progressive dystonia and parkinsonism during childhood [7]. Deficiency of mouse PanK2 leads to azoospermia but, unlike the human disease, there is no apparent neuromuscular dysfunction or brain iron accumulation [8]. The lack of correlation between the mouse PanK2-null phenotype and human PKAN disease is not yet understood.

**Figure 1:**
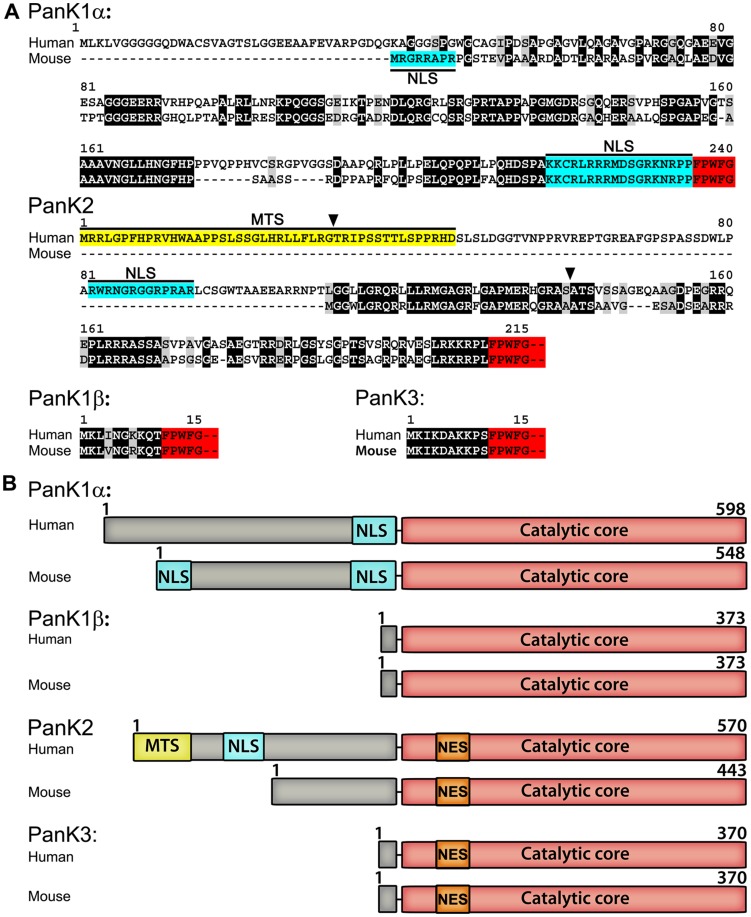
Alignment of the amino-terminal sequences of human and mouse PanK isoforms. (A) Identical (black) or conserved (gray) residues between the human and mouse isoforms are indicated. The amino acids corresponding to the beginning of the catalytic domain of each isoform are indicated in red. The mitochondrial targeting sequence (MTS) of hPanK2 is indicated in yellow, and the nuclear localization signals (NLS) are highlighted in cyan. (B) Schematic diagram of human and mouse full-length PanK proteins. The numbers indicate the total length of the PanK proteins. The results that follow demonstrate the functionality of nuclear localization signals (NLS) and nuclear export signals (NES), which are highlighted in cyan and orange, respectively.

There are four different active PanK proteins in humans and mice that share a common catalytic domain that is >80% identical (Fig. 1A and 1B). The PanK1β and PanK3 proteins are shorter than the PanK1α and PanK2, and have 10 residue amino-terminal extensions from their catalytic domains. The PanK1β and PanK3 protein sequences are highly homologous but possess distinct regulatory properties [9]. PanK3 is very sensitive to inhibition by long-chain acyl-CoAs, but PanK1β is not, and this difference in feedback regulation was previously mapped to regions within the catalytic domain of each protein [9]. PanK2 is most stringently regulated by acetyl-CoA [1]. Both PanK2 and PanK3 are activated by interaction with acyl-carnitines [10], which are metabolic intermediates that accumulate when the cell is overloaded with fatty acid, or acyl-ethanolamides [11] which are novel signaling molecules in the central and peripheral nervous system. Most cell types express several PanK isoforms, but PKAN disease and the PanK1 knockout mouse show that the loss of one PanK isoform is not always compensated by the expression of the other isoforms [4], [8]. Although the PanKs catalyze the same step in CoA biosynthesis, the differences in their N-terminal structures may direct the proteins to different cellular compartments to enable them to sense the need for CoA at these locations. The goal of this study was to determine the subcellular localization of each PanK isoform and define the molecular signals that direct them to these locations.

## Results

### Overview of PanK Localization

**Figure 2:**
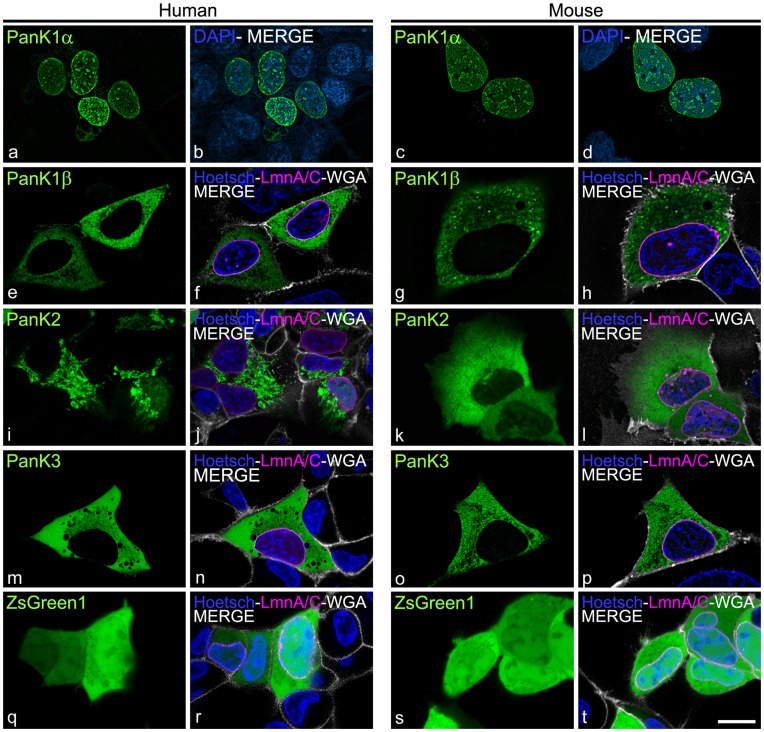
Distribution of human and mouse PanK isoforms. HEK293 cells were transfected with expression plasmids encoding hPanK1α-His (a, b), mPanK1α-His (c, d), hPanK1β-ZsGreen1 (e, f), mPanK1β-ZsGreen1 (g, h), hPanK2-ZsGreen1 (i, j), mPanK2-ZsGreen1 (k, l), hPanK3 (m, n), mPanK3 (o, p) and ZsGreen1 (q-t). PanK proteins are green, cell nuclei were stained with DAPI (blue, b, d) or Hoechst 33342 (blue, f, h, j, l, n, p, r, t). Cotransfection with an expression plasmid encoding Lamin A/C-mCherry (LmnA/C, magenta, f, h, j, l, n, p, r, t) designated nuclear membrane and staining with wheat germ agglutinin (WGA)-Alexa647 designated plasma membrane (white, f, h, j, l, n, p, r, t). The PanK1α constructs were visualized by immunocytochemistry in fixed cells using an anti-His tag antibody (a, b, c, d). The PanK1β, PanK2, PanK3 fluorescent fusion proteins as well as ZsGreen1 protein alone were visualized by live-cell confocal fluorescent microscopy. The results are representative of 2 or more independent experiments. The results for PanK2 were confirmed in a mouse cell line, NIH3T3 [5]. Scale bar, 10 µm.

The PanK1α proteins are the longest PanKs, having an amino-terminal extension of 235 or 185 amino acids for the human and mouse proteins, respectively (Fig. 1). The mouse isoforms are designated as mPanK and the human forms as hPanK. Alignment of the amino-terminal sequences of the human and mouse proteins share 85% identity. Stable cell lines expressing either human or mouse His-tagged PanK1α were visualized by immunocytochemistry using a monoclonal antibody that recognized the carboxy-terminal His-tag. Expression of human or mouse PanK1α fused to ZsGreen1 fluorescent protein in HEK293 cells resulted in fluorescent protein aggregates throughout the cell, compromising the interpretation of live-cell images. Both human and mouse PanK1α proteins were nuclear (Fig. 2, a–d). The full-length cDNAs for the other isoforms were fused to the cDNA encoding ZsGreen1 and protein expression was imaged in living cells. ZsGreen1 fluorescent protein is derived from *Zoanthus* species [12] and can be fused to a different protein without interfering with the normal localization [13]. ZsGreen1 has high solubility, extremely bright emission, and rapid chromophore maturation in mammalian cells [14], [15]. Both human and mouse PanK1β localized to the cytosol and exhibited a punctate pattern, suggesting a potential association with intracellular vesicles. (Fig. 2, e–h). The hPanK2 protein was detected in both mitochondrial and nuclear compartments (Fig. 2, i–j). Mouse PanK2 and both human and mouse PanK3 were cytosolic with a diffuse pattern, indicating that these proteins were soluble and not associated with structural components of the cell (Fig. 2, k–p). Finally, expression of ZsGreen1 alone showed a freely soluble protein that distributes throughout the cell (Fig. 2 q–t).

### PanK1α Localized to the Nucleus

**Figure 3:**
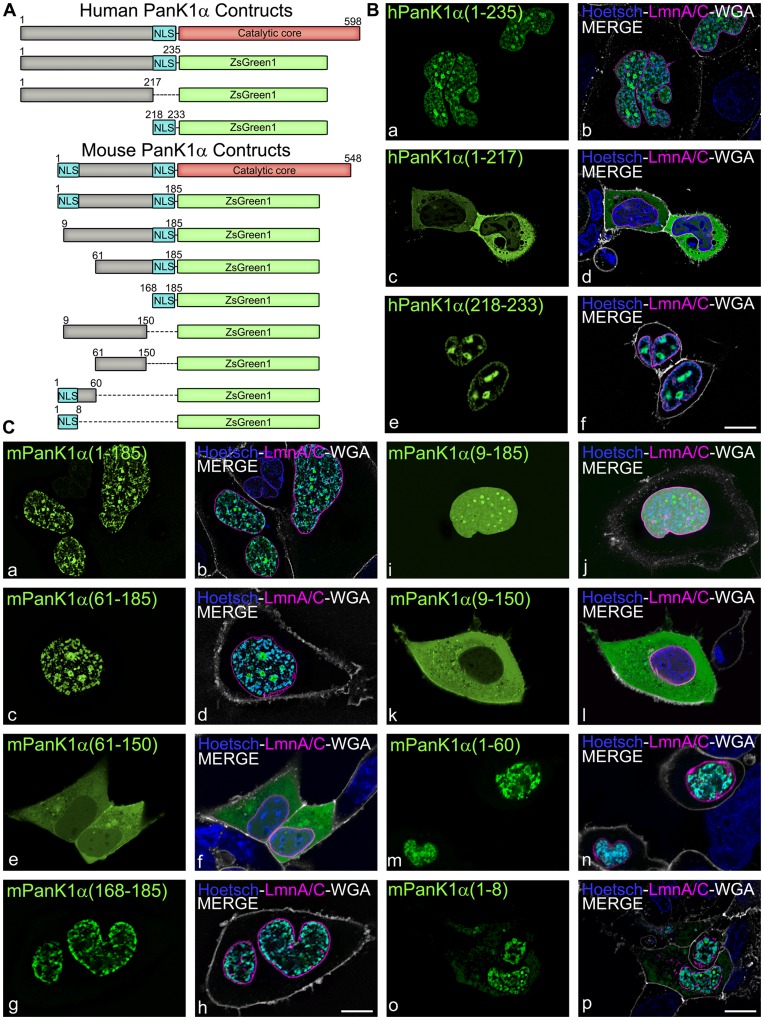
Identification of NLS in the human and mouse PanK1α isoforms. HEK293 cells were transfected with expression plasmids encoding PanK1α partial sequences fused to ZsGreen1. (A) Schematic diagram of human and mouse PanK1α proteins and their derivatives named: hPanK1α(1–235), hPanK1α(1–217), hPanK1α(218–233), mPanK1α(1–185), mPanK1α(9–185), mPanK1α(61–185), mPanK1α(168–185), mPanK1α(9–150), mPanK1α(61–150), mPanK1α(1–60) and mPanK1α(1–8). The numbers indicate amino acid positions included in the fusion proteins. Dashed lines represent internal deletions. The NLS are indicated in dark gray. (B and C) Functional analysis of predicted NLS of PanK1α. hPanK1α expression constructs (panel B, a–f, green) or mPanK1α constructs (panel C, a-p, green) were co-transfected with an expression plasmid encoding lamin A/C fused to mCherry (LmnA/C, magenta), which designates the nuclear membrane, and before imaging, cells were counterstained with wheat germ agglutinin (WGA)-Alexa 647 (white) and Hoetsch 33342 (blue) to visualize the plasma membrane and the nucleus, respectively. The results are representative of at least 2 independent experiments. Scale bar, 10 µm.

**Figure 4:**
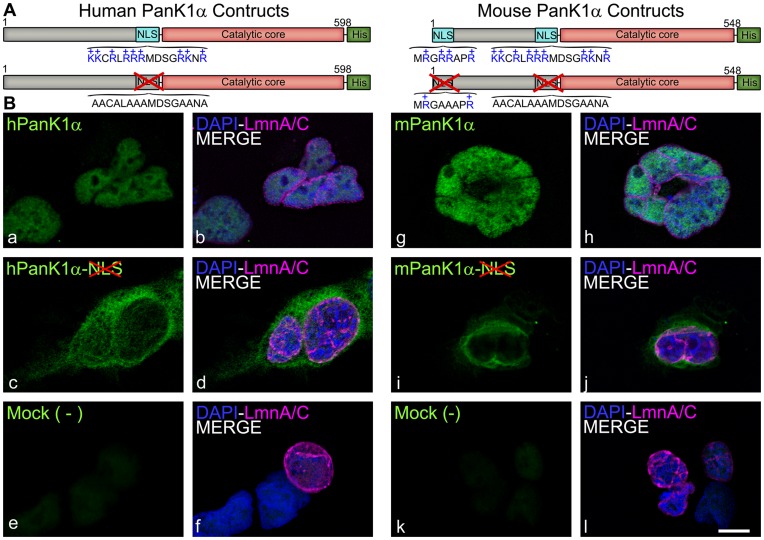
Mutagenesis of NLS in the human and mouse PanK1α isoforms. (A) Schematic diagram of human and mouse PanK1α proteins and their mutant derivatives named: hPanK1α-noNLS-His and mPanK1α-noNLS-His, containing the disrupted NLS motifs as indicated. (B) HEK293 cells were transfected with expression plasmids encoding wild type hPanK1α-His (a, b), mPanK1α-His (g, h), hPanK1α-noNLS-His (c, d), mPanK1α-noNLS-His (i, j). His-tagged PanK proteins are green, cell nuclei were stained with DAPI (blue, b, d, f, h, j, l). Cotransfection with an expression plasmid encoding Lamin A/C-mCherry (LmnA/C, magenta, d, b, d, f, h, j, l) designated nuclear membrane. The PanK1α constructs were visualized by immunocytochemistry in fixed cells using an anti-His tag antibody (a, c, g, i). Results are representative of at least 2 independent experiments. Scale bar, 10 µm.

**Figure 5:**
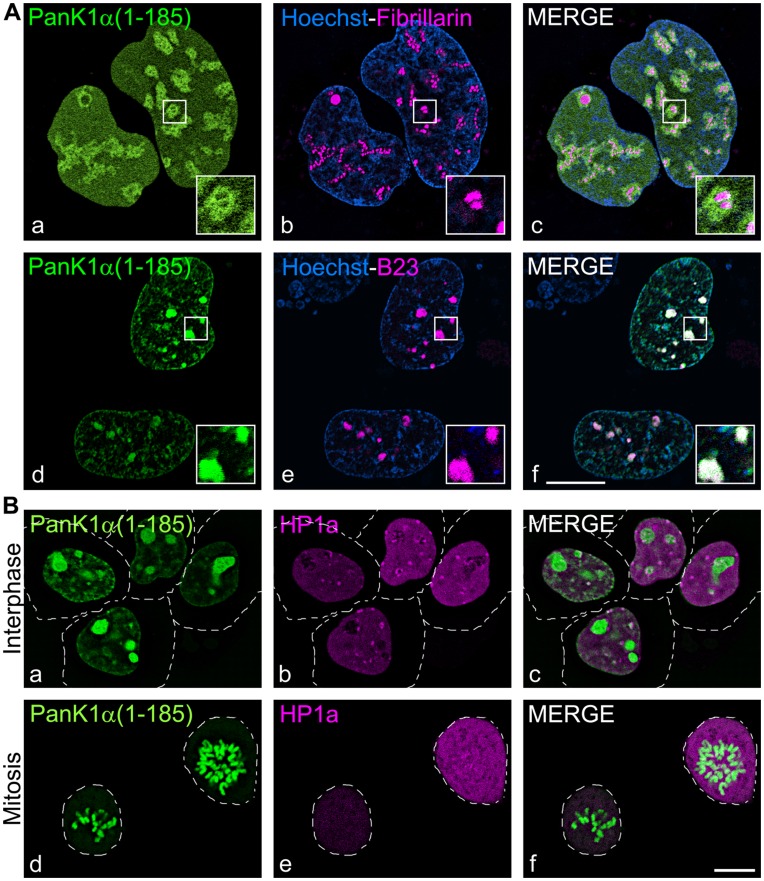
Localization of PanK1α within the nucleolus and with the perichromosomal region during mitosis. HEK293 cells were transfected with expression plasmids encoding mPanK1α(1–185) fused to ZsGreen1 (panels A and B, a, c, d, f, green). (A) Cells were co-transfected with plasmid pAA076 encoding human fibrillarin fused to mCherry (Fibrillarin, magenta, b, c) or plasmid pAA079 encoding human B23 fused to mCherry (B23, magenta, e, f). Fibrillarin designates the dense fibrillar component and B23 designates the granular component of nucleoli. Cell nuclei were visualized by staining with Hoechst 33342 (blue). Cells were visualized using live-cell confocal imaging. Co-localization in merged images are indicated by white pixels containing both magenta and green pseudocolored contributions. Insets in merged images show details at higher magnification. (B) HEK293 cells were co-transfected with with a plasmid encoding human heterochromatin protein 1α fused to mCherry (HP1a, b, c, e, f, magenta). Cells were visualized using live-cell confocal imaging. During interphase, mPanK1α associated with nucleoli and HP1a associated with relaxed heterochromatin, thus designating the nuclear region. In mitotic cells, the nuclear envelope is absent and mPanK1α associated with condensed chromosomes, while HP1a was dissipated throughout the cytoplasm. Dashed lines delimit cell borders (note that mitotic cells are spherical and detached from the substratum). Results are representative of at least 2 independent experiments. Results obtained using hPanK1α were the same. Scale bar, 10 µm.

Analysis of the PanK1α amino-terminal sequences by the Hidden Markov model for nuclear localization signal (NLS) prediction [16] revealed two putative nuclear localization signals within mouse PanK1α and one potential NLS within hPanK1α (Fig. 1A and 1B). Deletion mutants of human and mouse PanK1α containing different segments of the amino-termini fused to ZsGreen1 were made (Fig. 3A). The NLS of hPanK1α was located between residues 218 and 233, because this short peptide was sufficient to direct ZsGreen1 into the nucleus of HEK293 cells and constructs excluding this region were cytosolic (Fig. 3B, a–f). mPanK1α contained the identical sequence between residues 168–185, and this sequence also directed the fusion protein to the nucleus (Fig. 3C, g, h). In addition, residues 1–8 of mPanK1α localized most of the ZsGreen1 fusion protein to the nucleus (Fig. 3C, o, p). We confirmed that the NLS motifs were required for the proper localization of the PanK1α proteins by substituting the positively charged amino acids lysine (K) and arginine (R) with alanine (A) by site directed mutagenesis in the NLS sequence (Fig. 4A). HEK293 cells were transfected with plasmids encoding His-tagged full-length proteins containing the mutated motifs and the expressed proteins were localized by immunocytochemistry using the monoclonal antibody that recognized the His-tag (Fig. 4B). hPanK1α with the mutated NLS was found primarily in the cytoplasm, with a fraction of the protein located in the nucleus as well (Fig. 4B, c, d). Mutated mPanK1α was found outside of the nucleus (Fig. 4B, i, j). Mock transfected cells did not react with the antibody (Fig. 4B, e–k). The unmutated PanK1α constructs were not evenly distributed within the nucleus, suggesting association with heterochromatin (designated by Hoechst staining), and an association with subnuclear structures that appeared to be nucleoli. Fluorescent markers for two of the three main components of the nucleolus were constructed to investigate these potential associations. B23-mCherry decorates the granular component (GC), and fibrillarin-mCherry decorates the dense fibrillar component (DFC) [17]. These markers were co-transfected in HEK293 cells with the fluorescent mPanK1α(1–185)-ZsGreen1 plasmid and visualized by live cell confocal microscopy. The PanK1α construct surrounded but did not co-localize with the DFC (Fig. 5A, a–c). In contrast, the PanK1α construct co-localized with the B23-mCherry construct, which designated the GC (Fig. 5A, d–f), as shown by white pixels resulting from both magenta and green pseudocolored contributions. The fluorescent PanK1α construct, similar to many other nucleolar proteins including B23, became disassociated from the nucleoli and associated with the perichromosomal region in mitotic cells (Fig. 5B, d–f). The heterochromatin protein 1a (HP1a) typically dissociates from chromosomes during mitosis and remains dissociated until the end of this process. This marker was included to better distinguish the location of the PanK1α construct during interphase and mitosis. The perichromosomal layer contains many proteins required for a variety of cellular processes but its role is not fully defined and remains controversial [18]. The localization of PanK1α in the nucleus and PanK1β in the cytosol makes an important distinction between these proteins that was missed in earlier work [19]. The previous interpretation was based on a correlation between increased hPanK1α transcript abundance and increased cytosolic staining with an antibody that recognized the common PanK1 catalytic domain. We now know that cytosolic PanK1β is very abundant in hepatocytes compared to PanK1α [4], and the majority of the PanK1 was cytosolic PanK1β rather than the minor nuclear PanK1α component.

### Cytosolic PanK1β Associated with Vesicles

**Figure 6:**
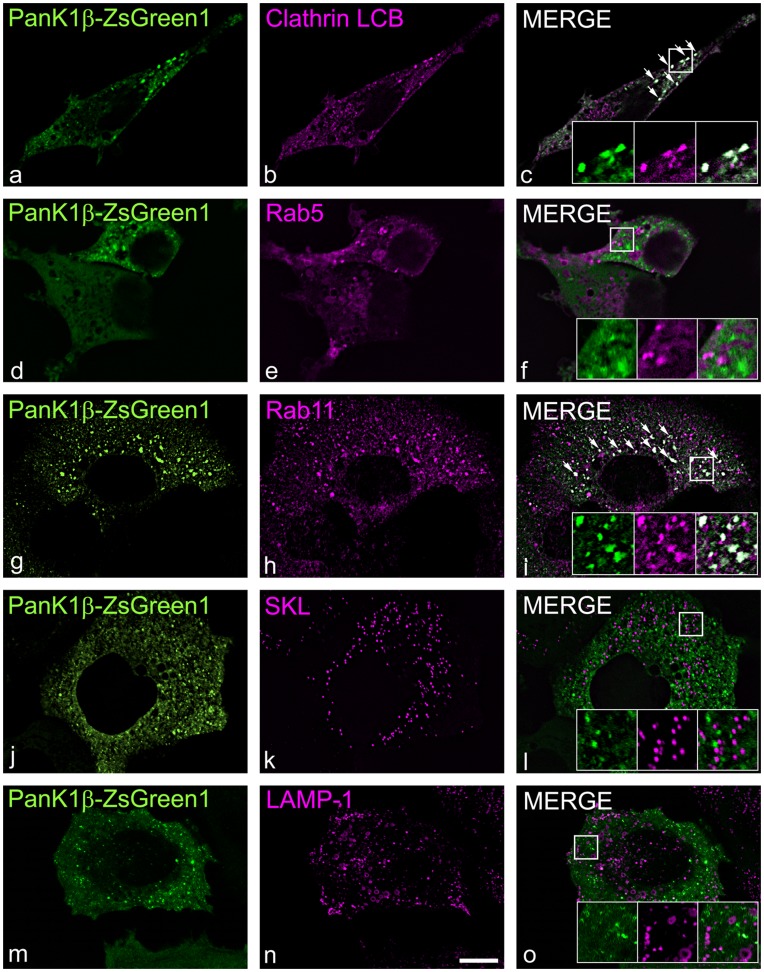
Cytosolic PanK1β partially associates with clathrin-coated and recycling endosomes. HEK293 cells were transfected with expression plasmids encoding mPanK1β fused to ZsGreen1 (green, a, c, d, f, g, i, j, l, m, o), together with different plasmids encoding the fluorescent protein mCherry fused to distinct markers for different subcellular vesicles: human clathrin LCB (magenta, b, c), human Rab5 (magenta, e, f), human Rab11 (magenta, h, i), firefly luciferase peroxisomal targeting signal (magenta, k, l) and rat Lamp1 (magenta, n, o). Rab5 designates early endosomes, Rab11 designates recycling endosomes and Lamp1 designates lysosomes. Cells were visualized using live-cell confocal imaging. Merged images show co-localization as indicated by white pixels resulting from both magenta and green pseudocolored contributions. Insets in merged images show details at higher magnification. Results are representative of 2 or more independent experiments. Scale bar, 10 µm.

Both human and mouse PanK1β localized to the cytosol and exhibited a punctate pattern, suggesting an association with intracellular vesicles (Fig. 2, e–h). To investigate which classification of vesicles may be associated with PanK1β, fluorescent protein markers that identify different vesicles types were co-transfected with the PanK1β constructs and co-localization was assessed in HEK293 cells. A portion of PanK1β associated with clathrin-coated structures (Fig. 6, a–c) and the localization of another portion of PanK1β with recycling endosomes was indicated by co-localization with the marker Rab11 GTPase (Fig. 6, g, h). In contrast, PanK1β was not associated with early endosomes, peroxisomes or lysosomes as evidenced by the lack of co-localization with the specific markers Rab5 (Fig. 6, d–f), SKL (Fig. 6, j–l), or Lamp1 (Fig. 6, m–o), respectively. These data place PanK1β from both species in the cytosol with some of the proteins associated with clathrin-coated structures and recycling endosomes.

### Human PanK2 Has a Nuclear Localization Signal

**Figure 7:**
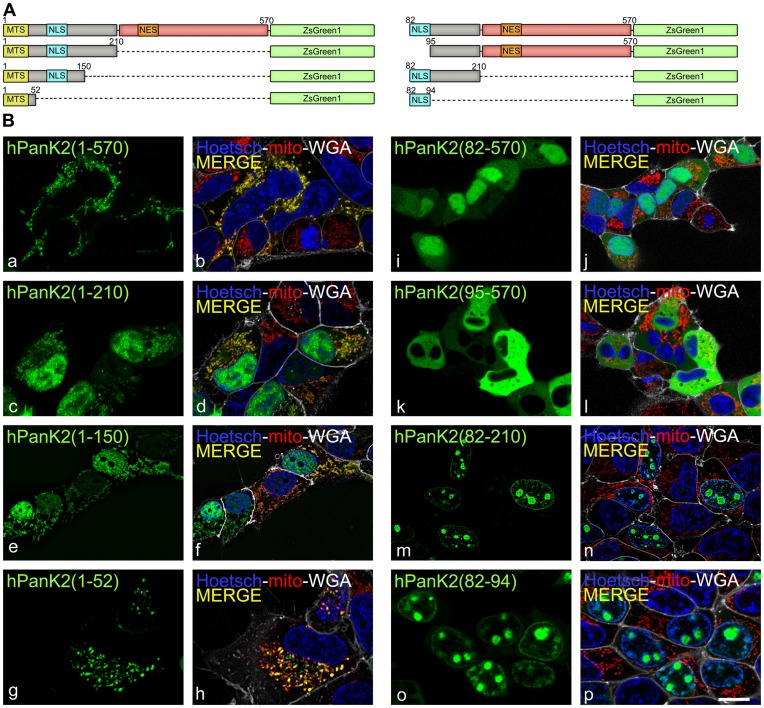
Identification of the NLS of human PanK2. (A) Schematic diagram of the full length hPanK2(1–570) and derivatives named hPanK2(1–210), hPanK2(1–150), hPanK2(1–52), hPanK2(82–570), hPanK2(95–570), hPanK2(82–210), and hPanK2(82–94) fused with ZsGreen1. The indicated numbers are the amino acid residues encoded by the constructs. (B) HEK293 cells were transfected with the indicated hPanK2 sequences fused with ZsGreen1 and the fusion proteins (green) were visualized by live cell confocal microscopy. Cells were counterstained with MitoTracker Red CMXRos (mito, red), WGA-Alexa 647 (white) and Hoetsch 33342 (blue) to visualize mitochondria, plasma membrane and nuclei, respectively. Merged images (b, d, f, h, j, l, n, p) show the co-localization indicated by yellow pixels containing both red and green pseudocolored contributions. Results are representative of at least 2 experiments. Scale bar, 10 µm.

**Figure 8:**
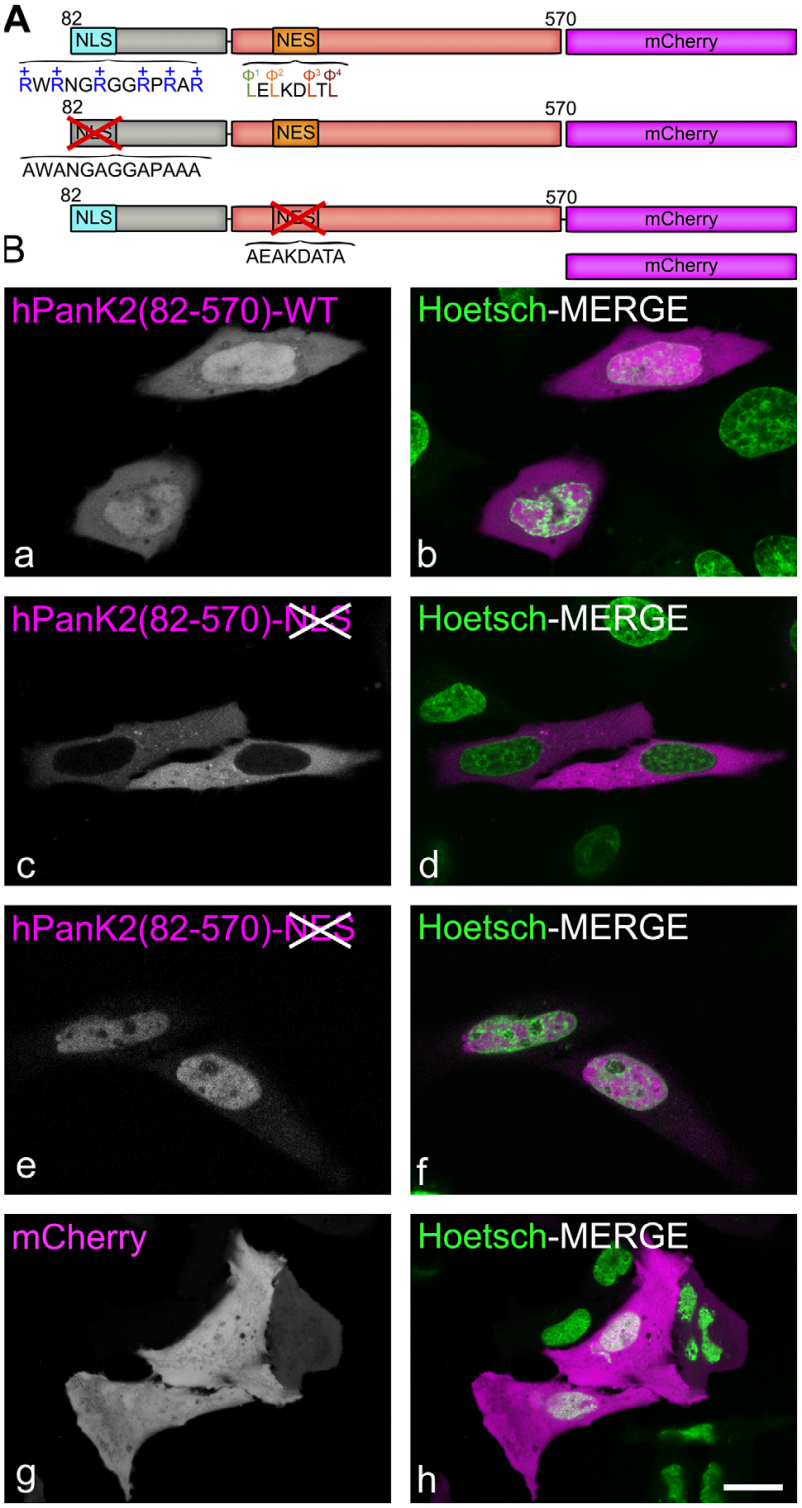
Mutagenesis of the NLS and NES of human PanK2(82–570). (A) Schematic diagram of human PanK2 without the MTS and the mutant derivatives named: hPanK2-noNLS-mCherry and hPanK2-noNES-mCherry, each containing disrupted NLS or NES motifs, respectively. Arginine (R) or leucine (L) residues were replaced by the alanine (A) as indicated. (B) HeLa cells were transfected with expression plasmids encoding hPanK2(82–570)-mCherry (a, b), hPank2(82-570-noNLS)-mCherry (c, d), hPanK2(82-570-noNES)-mCherry (e, f) or mCherry alone (g,h). After 48 hours cells were stained with Hoetsch 33342 to visualize nuclei (green, b, d, f, h) and analyzed by live-cell confocal microscopy. Results are representative of at least 2 independent experiments. Scale bar, 10 µm.

PanK2 proteins from human and mouse species share a highly homologous catalytic domain with similar biochemical properties [5], but the amino-terminal domains are substantially different (Figure 1). It was previously reported that human PanK2 was a mitochondrial protein [20]–[22], whereas mPanK2 was diffusely distributed in the cytosol [5] (Fig. 2k). The location of hPanK2 was investigated using a series of truncated constructs encoding different segments of the amino-terminus fused to ZsGreen1 (Fig. 7A). HEK293 cells were transfected with these constructs and later counterstained with fluorescent markers for plasma membrane (wheat germ agglutinin, WGA-Alexa 647), mitochondria (MitoTracker® Red CMXRos) and nuclear heterochromatin (Hoechst 33342), and visualized by live-cell confocal microscopy. As expected, all fusion proteins with the mitochondrial targeting signal (MTS) from amino acids 1–46, were associated with mitochondria as shown by white pixels resulting from co-localization of ZsGreen1 with MitoTracker® Red (Fig. 7B, b, d, f, h). In addition, the fusion protein containing the complete amino terminus of human PanK2 (1–210)-ZsGreen1 was found in both the nucleus and mitochondria (Fig. 7B, c and d, Fig. S1). Two predicted NLS sequences were postulated to exist between amino acids 162–168 and 205–209 [23], but when these segments were removed from the hPanK2(1–150) fusion proteins, the distribution between the nuclear and mitochondrial compartments was unchanged (Fig. 7B, e, f). These data suggested that a functional NLS in PanK2 was located between the MTS and residue 150 (Fig. 7A). Accordingly, the basic sequence “RWRNGRGGRPRAR” located between residues 82 and 94 was identified and found to be sufficient to translocate the ZsGreen1 fusion protein to the nucleus (Fig. 7B, o, p). The construct hPanK2(95–570) that excluded this 13-residue sequence did not encode a nuclear protein (Fig. 7B, k, l). Thus, the sequence from residues 82–94 that are unique to hPanK2 contained the only NLS. The functionality of the NLS in the context of the larger hPanK2 protein was confirmed by mutating the 6 arginine (R) residues from residues 82–94 to alanine (A) in hPanK2(82–570) (Fig. 8A). The mutated protein was fused to the fluorophore mCherry and visualized by live-cell confocal microscopy (Fig. 8B a–d). mCherry is a monomeric red fluorescent protein with improved brightness and photostability compared to the prototypic DsRed protein [24]. Mutation of the NLS prevented the nuclear localization of the fluorescent fusion protein, confirming that this motif was required for translocation. Interestingly, full-length hPanK2, including the mitochondrial targeting sequence and the catalytic domain, was associated predominantly with mitochondria and its presence in the nucleus was less frequent (Fig. 2B and Fig. S1). This observation suggested the existence of a nuclear export signal (NES) sequence within the catalytic domain.

### Human PanK2 Has a Nuclear Export Signal

**Figure 9:**
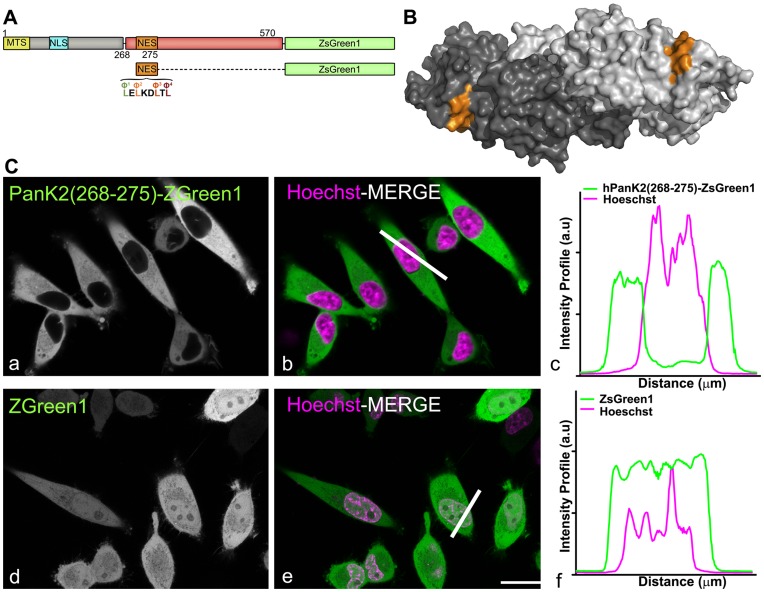
Human PanK2 has a functional NES. (A) Schematic diagram of full length hPanK2 highlighting the mitochondrial targeting signal (MTS, yellow), nuclear localization signal (NLS, cyan) and the nuclear export signal (NES, orange). The CRM1-NES consensus sequence included residues 268–275 of hPanK2 which were fused with ZsGreen1. Amino acid sequence of the hPanK2 NES is shown, consisting of four bulky hydrophobic amino acids (indicated by Φ1–Φ4) plus three charged residues. (B) Crystal structure of the human PanK3 dimer with each monomer shown in a different shade of gray, and the nuclear export signal LELKDLTL shown in orange on the surface of each monomer. (C) HeLa cells were transfected with the expression plasmid pAA303 encoding the hPanK2(268–275) fused to ZsGreen1 or with the plasmid encoding ZsGreen1 only. Cells were counterstained with Hoetsch 33342 (magenta); to visualize nuclei and treated with cycloheximide (50 µg/ml) to inhibit de novo protein synthesis. White lines (panels b and e) indicate the regions of each image that were scanned for distribution of fluorescence, shown in panels c and f (magenta, Hoechst 33342, and green, ZsGreen1). Scale bar, 10 µm.

A bioinformatics analysis [25] predicted that a highly probable leucine-rich CRM1-dependent NES may exist in both mouse and human PanK2, located between residues 268–275 of the hPanK2 catalytic domain (Fig. 9A). This sequence, LELKDLTL, contains four hydrophobic residues (indicated as Φ^1^–Φ^4^) and two negatively charged acidic residues (Fig. 9A). The same sequence is located within the highly homologous catalytic domain of hPanK3 for which a structure is available [26]. The NES motif is located on the surface of the protein which is sufficiently exposed to facilitate interaction with the nuclear export machinery (Fig. 9B). This potential NES of PanK2 was fused with fluorescent ZsGreen1 protein (Fig. 9A) and the subcellular localization of the expressed protein encoded by the construct was determined. Coupling the 8 amino acids of the putative NES to the amino-terminus of ZsGreen1 (PanK2(268–275)-ZsGreen1) targeted the reporter protein to the cytoplasm (Fig. 9C, a–c), whereas the ZsGreen1 protein alone was uniformly distributed between the nucleus and the cytoplasm (Fig. 9C, d–f and Fig. 2 q–t). Quantification of over 100 cells confirmed these findings (Fig. S2). These results indicated that both ZsGreen1 and PanK2(268–275)-ZsGreen1 proteins were able to diffuse into nuclei, but only the construct containing the NES (PanK2(268–275)-ZsGreen1) was actively excluded from the nucleus.

**Figure 10:**
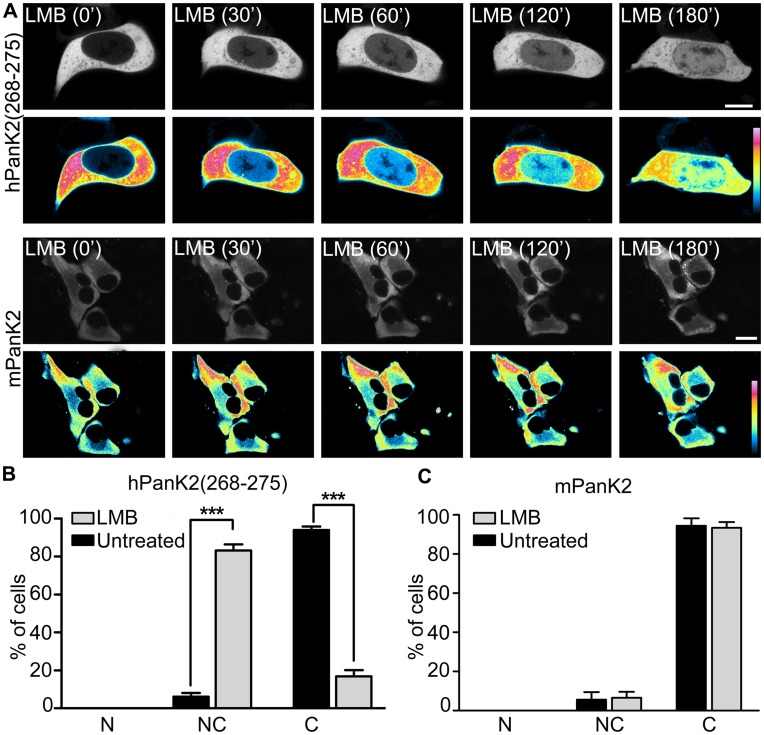
Leptomycin B blocks the nuclear export of human PanK2. (A) HeLa cells were transfected with the expression plasmid pAA303 encoding hPanK2(268–275) fused to ZsGreen1, or with plasmid pAA338 encoding the full length mPanK2 fused to ZsGreen1. Leptomycin B (LMB) (20 nM) and cycloheximide (50 µg/ml) were added 24 hours later. Fluorescent cells were imaged at times indicated (upper panels, black and white). Pseudo-colored micrographs are shown to better visualize the fluorescence intensity levels (lower panel, color). (B). Scoring of fluorescent cells (LMB, n = 249 cells; Control, n = 284 cells) for subcellular distribution of hPanK2(268–275)-ZsGreen1 and (C) scoring of fluorescent cells (LMB, n = 129 cells; Control, n = 143 cells) for subcellular distribution of mPanK2-ZsGreen1 after LMB treatment as nuclear “N”, [nuclear and cytoplasmic] “NC” or cytoplasmic “C”. Significance was determined using unpaired Students t-test. ***p<0.001. Scale bar, 10 um.

NES sequences are recognized by a soluble export receptor CRM1 (also called Exportin1) that mediates formation of a trimeric complex between the protein carrying the NES, CRM1 and Ran-GTP. The trimeric complex is transported out of the nucleus into the cytoplasm, where it dissociates and releases the protein in the cytosol [27]. Leptomycin B (LMB) is a specific inhibitor of CRM1-mediated nuclear export [28], [29]. Time-lapse experiments using the fluorescent construct PanK2(268–275)-ZsGreen1 were performed after the addition of LMB to determine if the protein exit was CRM-1 dependent. When the nuclear export process was interrupted with LMB treatment, the fluorescence intensity of hPanK2(268–275)-ZsGreen1 in the nucleus increased, while the cytoplasmic fluorescence diminished (Fig. 10A, upper white and lower pseudocolored images). These data indicated that the hPanK2(268–275)-ZsGreen1 construct was retained in the nucleus and quantification of >200 cells confirmed that the translocation from nucleus to cytoplasm was CRM1-dependent (Fig. 10B). The mPanK2 protein lacks a NLS (Fig. 1A and 1B) [5], but has the identical NES sequence identified in hPanK2 (Fig. 1B). When mPanK2 was fused to ZsGreen1, the fluorescent protein was not detected in the nucleus (Fig. 2, k-i), even after LMB treatment which would inhibit CRM1-mediated nuclear export (Fig. 10A). Quantification of >120 cells confirmed that the subcellular distribution of mPanK2-ZsGreen1 was not affected by LMB treatment (Fig. 10C). Cells were viable up to 8 hours after treatment (Fig. S3 C,D). In comparison, the PanK1α and PanK1β proteins contain NLS motifs and do not contain intact NES sequences, because the most critical residue in the NES sequence - the fourth leucine (L) - is replaced by a methionine (M), thus enabling the import and retention of these proteins in the nuclear compartment. The hPanK2(82–570) protein corresponding to the full-length PanK2 without the MTS and including the NLS and the NES was fused to the mCherry fluorescent protein (hPank2(82–570)-mCherry) to confirm that the NES was functional in the context of the larger protein (Fig. 8A and 8B, e, f). The wild-type (WT) hPanK2(82–570)-mCherry fusion protein was found both in the nucleus and cytoplasm, with a stronger nuclear signal. The hPanK2(82–570)-mCherry fusion protein with the mutated NES was found in the nucleus only (Fig. 8 e, f). Inhibition of nuclear export by LMB treatment caused the cytoplasmic fluorescence intensity to diminish to almost background levels (Fig. S3A). Quantification of >200 cells indicated that the nucleo-cytoplasmic distribution of this PanK2 fusion protein was perturbed by LMB inhibition (Fig. S3B). These data demonstrated that both hPanK2 and mPanK2 contain sequences that direct export of the proteins from the nucleus.

### Human PanK2 Migrated from Nucleus to Mitochondria in a Cell Cycle–Dependent Manner

**Figure 11:**
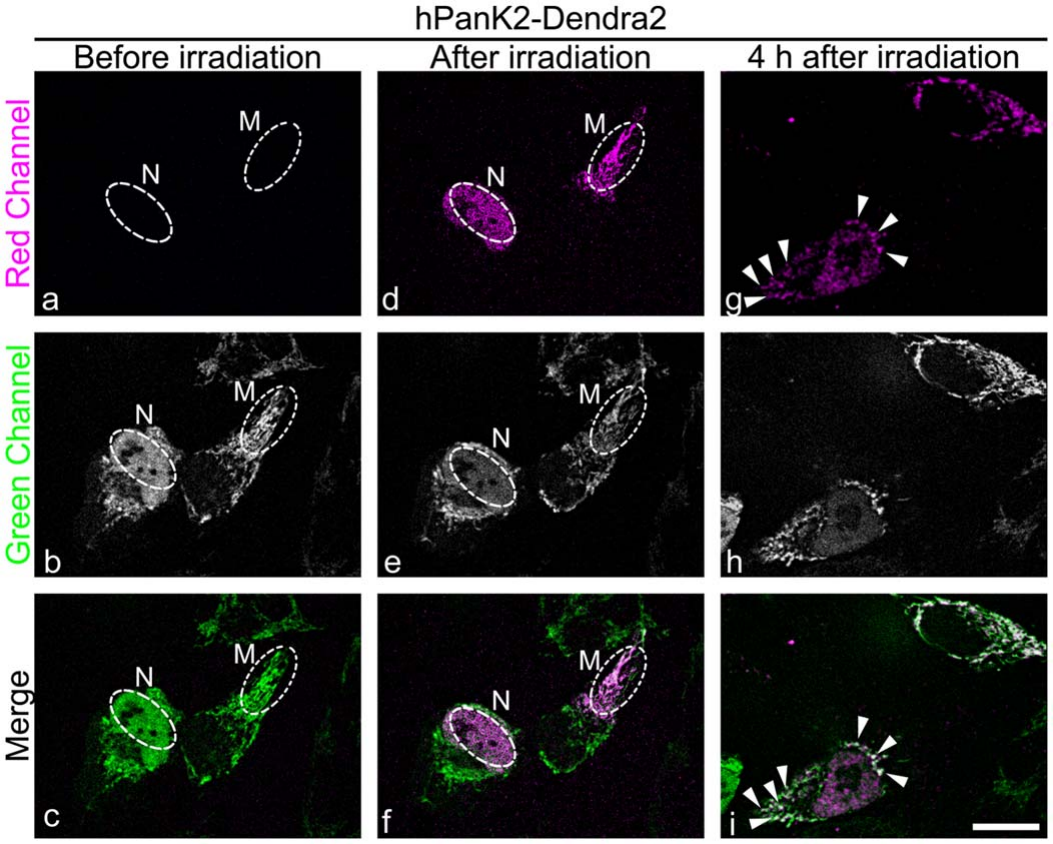
Human PanK2 migrates from nucleus to mitochondria. HeLa cells were transfected with the expression plasmid pAA275 encoding full-length hPanK2(1–570) fused to the green-to-red photoswitchable fluorescent protein Dendra2. The red-irreversibly-converted fluorescence was real-time tracked in living cells. The micrographs show two cells (before irradiation, panels a, b, c) expressing hPanK2-Dendra2, one cell showing “nuclear and mitochondrial” fluorescence and a second cell with “mitochondrial only” fluorescence. Dendra2 was selectively photoconverted within the indicated ROIs (white dashed ovals) for each cell (after irradiation, panels d, e, f) using brief illumination with 405 nm irradiation, and the fusion proteins were visualized in the green and red channels thereafter. After 4 hours (panels g, h, i), the photoconverted nuclear fraction of hPanK2 was associated with mitochondria (arrows, panels g, i) whereas the photoconverted mitochondrial hPanK2 in the other cell remained associated with mitochondria during the same time. Co-localization of both magenta and green hPanK2 fusion proteins associated with mitochondria are indicated by white pixels and arrows in the merged image (panel i). Scale bar, 10 µm.

The presence of hPanK2 in both the nucleus and mitochondria raised the question as to whether the protein was exchanged between these two compartments of the cell. Cells were transfected with the full-length hPanK2 (1–570) fused to the photoactivatable protein Dendra2. Dendra2 protein undergoes an irreversible photoconversion from green to red fluorescence after intense-blue-light irradiation, which enables the tracking of specific pools of fusion proteins. Cells expressing the hPanK2-Dendra fusion protein in either the mitochondrial or [nuclear plus mitochondrial] compartments were irradiated within the indicated regions of interest (Fig. 11, Regions “M” and “N” a-f) and visualized four hours later (Fig. 11, g-i). The nuclear red fluorescence exited from the nucleus and became associated with mitochondria. The mitochondrial red fluorescence remained associated with mitochondria during the same period of time (Fig. 11 g, h, i), and for up to 12 hours later (data not shown), indicating that the mitochondria were the final destination for the protein. These data were consistent with the fact that the amino terminus of hPanK2 is truncated at residue 141 during mitochondrial entry [5], [20], [21], which would remove the hPanK2 NLS as well as the MTS.

**Figure 12:**
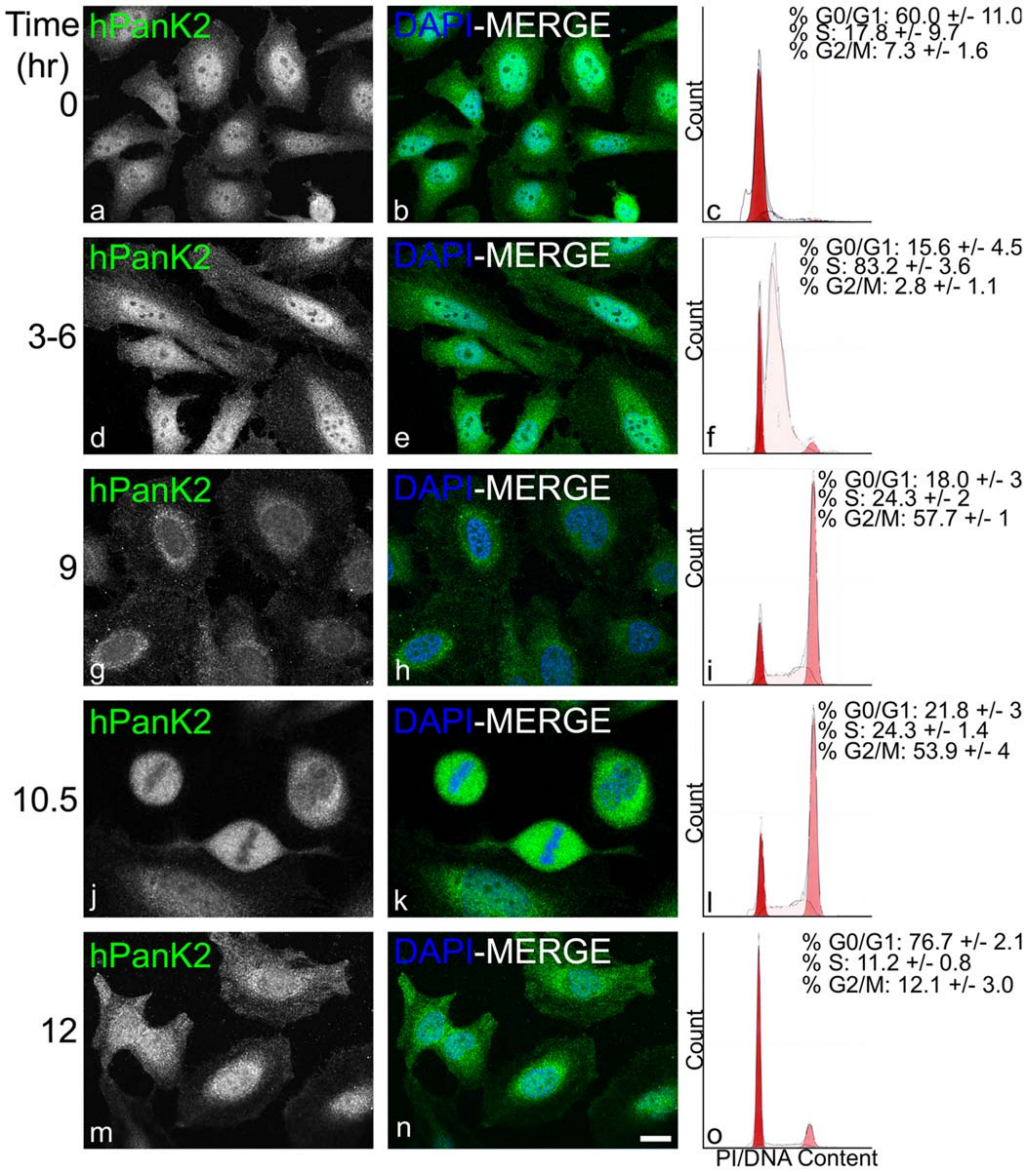
Dynamics of human PanK2 translocation during cell cycle progression. Synchronized Hela cells were released from a double thymidine block and immunostained with an anti-hPanK2 antibody to visualize hPanK2 (white, a, d, g, j and m; green, b, e, h, k, n) and heterochromatin (DAPI, blue; b, e, h, k and n) using fluorescent confocal microscopy. Representative images are shown at indicated times after release. Cell cycle distribution after release was determined in parallel experiments following propidium iodide staining of nuclear DNA and flow cytometry and representative data are shown (c, f, i, l, and o). The percentage of the cell population in each cell cycle phase was calculated using ModFit software at each time point (insets, c, f, I, l, o) and the average values ± s.e. from three independent experiments are indicated. Scale bar, 20 µm.

Cells with and without nuclear hPanK2 were observed in the same asynchronous population. We next investigated whether the subcellular distribution of hPanK2 was dependent on the cell cycle. Cells were arrested at the boundary between G1 and S phases using a double thymidine block [30] and then released. Following release, cells were fixed and immunostained to visualize the subcellular distribution of the endogenous hPanK2 protein at the times indicated in Figure 12. In a parallel separate experiment, cells were stained with propidium iodide to quantify the DNA content and analyzed by flow cytometry to monitor the progression of the population through the cell cycle after release (Fig. 12, c, f, i, I, o). The data indicated hPanK2 was present in both the nuclear and mitochondrial compartments during S phase, and then exited the nucleus during G2 phase, shortly before mitosis. At 10.5 hours after the release, the cells entered mitosis, the nuclear membrane disappeared, the chromatin was condensed and the hPanK2 proteins were visualized in both cytoplasmic and mitochondrial compartments. Upon reassemby of the nuclear membrane after mitosis, hPanK2 was again found in both the nuclear and mitochondrial compartments, as well as after cytokinesis and during the G1 phase of a subsequent cycle. The amount of nuclear hPanK2 was quantified using image analysis software and the results confirmed that at 9 hrs, which correlated with G2 phase, there was a significant reduction in nuclear hPanK2 signal (Fig. S4). These data indicated that hPanK2 was in the nucleus when DNA synthesis and gene expression were active and the chromatin was structurally loose. The hPanK2 was not found in the nucleus when the chromatin was preparing to become more tightly packaged and relatively inactive.

### Human PanK2 is in the Intermembrane Space of Mitochondria

**Figure 13:**
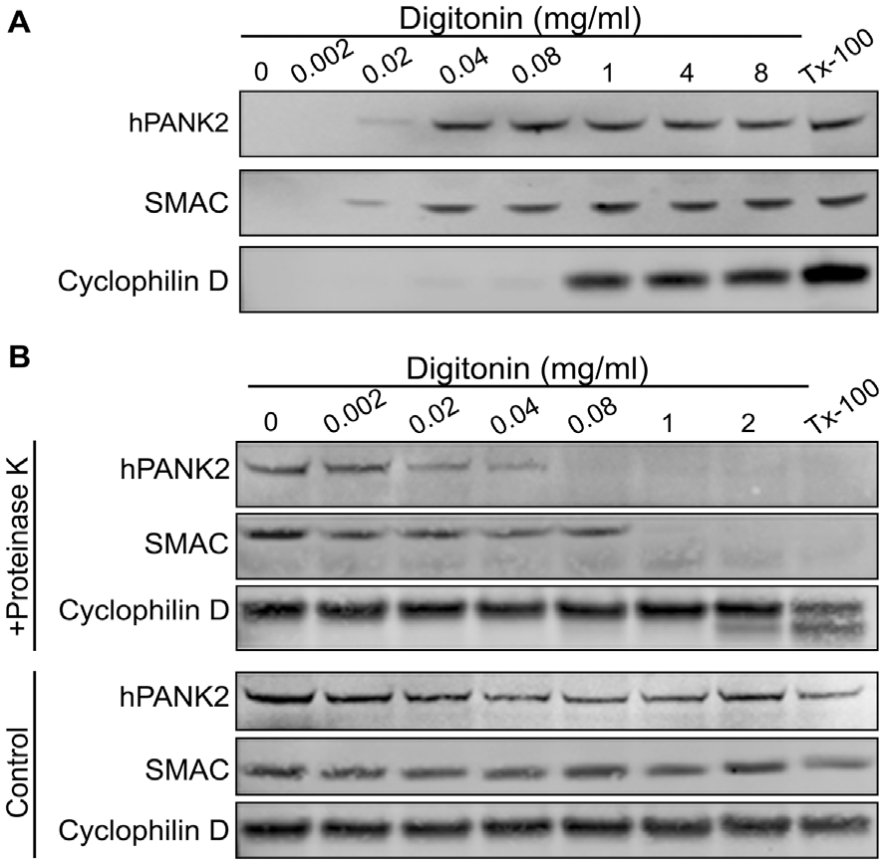
The mature hPanK2 protein is localized in the intermembrane space (IMS) of mitochondria. (A) SH-SY5Y cells, which express relatively high levels of hPanK2, were permeabilized with increasing concentrations of digitonin (0.002–8 mg/ml) or with TritonX-100 (0.1%) as indicated and samples were centrifuged as described in Materials and Methods. The supernatants were fractionated by SDS-PAGE, transferred to PVDF membranes and immunoblotting was performed using antibodies raised against hPanK2, SMAC/DIABLO (to designate IMS) and cyclophilin D (to designate mitochondrial matrix). (B) Intact SH-SY5Y cells were incubated with or without Proteinase K (50 µg/ml) together with increasing concentrations of digitonin (0.002–2 mg/ml) or with TritonX-100 (0.1%) as indicated. The samples were centrifuged and the pellets were fractionated by SDS-PAGE, transferred to PVDF membranes and immunobloted to detect hPanK2, SMAC/DIABLO (to designate IMS) and cyclophilin D (to designate mitochondrial matrix). Results are representative of three independent experiments.

Mitochondrial proteins are distributed in different compartments that carry out specialized functions. The major compartments are the outer membrane, the intermembrane space (IMS), the inner membrane, and the matrix. Although hPanK2 association with mitochondria is established [5], [21], [22], the precise localization of hPanK2 within the mitochondrial compartment is not known. We proposed that hPanK2 may be located in the IMS to enable interaction with long chain acyl-carnitines at their site of synthesis and to allow exit of the phosphorylated product, phosphopantothenate, to the cytosol where the downstream enzymes of CoA biosynthesis are located [10]. hPanK2 is processed by proteolytic cleavage twice during mitochondrial entry to a mature size of about 48 kDa [21] and mature hPanK2 is a soluble protein [10], [26], [31]. A biochemical approach based on selective permeabilization of the outer membrane by detergent, combined with protease protection assays, was used to investigate the submitochondrial localization of hPanK2 in the human neuroblastoma SH-SY5Y cell line [32]. These cells are frequently used as a model to study mechanisms of neuronal differentiation and function [33]–[35]. Intact cells were treated with increasing concentrations of digitonin to sequentially permeabilize the plasma membrane first, the outer mitochondrial membrane next and then the inner mitochondrial membrane at the highest concentrations. Complete solubilization of membranes was accomplished with Triton X-100 (Fig. 13). The permeabilized cells were separated into supernatant and pellet fractions by centrifugation, and the supernatants were fractionated by SDS-PAGE and hPanK2 and marker proteins identified by Western blot (Fig. 13A). hPanK2 and the IMS marker SMAC (second mitochondria-derived activator of caspases) were released into the supernatant by low digitonin (0.02 mg/ml) in contrast to the matrix protein, cyclophilin D which was not released until digitonin concentrations were >1 mg/ml. Treatment with Triton X-100 (0.1%) solubilized all of the mitochondrial contents and was used as a control condition. In a separate series of experiments, the pellet fractions containing the proteins remaining with the treated mitochondria were subjected to digestion with proteinase K (Fig. 13B). The pellet proteins were collected, separated by SDS-PAGE and identified by immunoblotting. hPanK2 and the marker proteins were recovered in the pellet fraction without digitonin treatment, both with and without proteinase K incubation (Fig. 13B). These data indicated that the mature processed hPanK2 was not accessible to the outside of mitochondria. The amounts of hPanK2 retained in the mitochondria, without protease, decreased as a function of increasing digitonin (Fig. 13B), corroborating the previous release experiment (Fig. 13A). Reduction in the amount of hPanK2 was observed following protease treatment of the 0.02 mg/ml digitonin pellet, and complete degradation occurred at 0.08 mg/ml digitonin. SMAC was completely degraded at 1 mg/ml digitonin. Proteolytic degradation of cyclophilin D became evident only at 2 mg/ml digitonin or upon complete disruption of mitochondria with Triton X-100. Together, these data supported the conclusion that the mature hPanK2 protein was located in the IMS of mitochondria.

## Discussion

**Figure 14:**
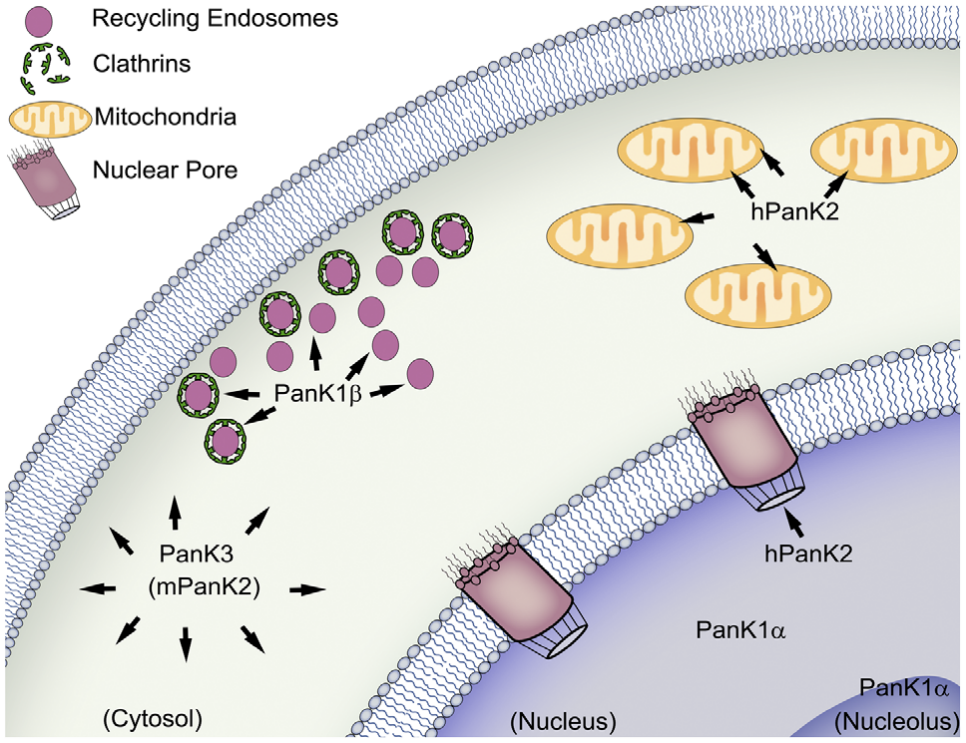
Subcellular distribution of PanK isoforms. PanK1α is sequestered in the nucleus, with preferential association within the nucleolus. PanK1β associates with clathrin-coated vesicles and recycling endosomes. Human PanK2 (hPanK2) is distributed in both the nucleus and the mitochondria. Nuclear hPanK2 translocates from nucleus to the intermembrane space of mitochondria. Mouse PanK2 (mPanK2) is cytosolic. PanK3 is distributed throughout the cytoplasm.

The different subcellular localizations for the PanK isoforms determined in this study are summarized in Fig. 14. PanK1α from both human and mouse species is sequestered in the nucleus, with preferential association within the nucleolus. PanK1β from both human and mouse associates with clathrin-coated vesicles and recycling endosomes. Human PanK2 is distributed in both the nucleus and the mitochondria, and the nuclear hPanK2 translocates from the nucleus to the intermembrane space of mitochondria. hPanK2 is found in both the mitochondria and the nucleus throughout the cell cycle, with the exception of the G2/M phase when hPanK2 is restricted to mitochdondria. Prior residence in the nucleus is not required for hPanK2 to be imported into mitochondria, as shown by the distribution of fusion proteins lacking the NES in both nuclear and mitochondrial compartments (Fig. S1). hPanK2 translocates from the nucleus to the mitochondria only (Fig. 11) due to the fact that the NLS is present in the full-length protein but not in the processed mitochondrial protein, which is cleaved at residue 141. hPank2 was previously detected in the nucleus following heterologous expression in yeast [23], but the NLS sequence predicted in that study is not functional. Mouse PanK2 is found in the cytoplasm exclusively, similar to human and mouse PanK3. This report for the first time describes the subcellular distribution of all the active PanK isoforms, and in particular, the transient nuclear localization of hPanK2. However, it is still unknown whether the nuclear localization of hPanK2 is required for a specific cellular function.

Although the PanK enzymes control the cellular CoA levels [1], [36]–[41], CoA synthesis does not occur at the locations of the PanKs. Rather, CoA is synthesized by a pathway initiated by PanK followed by three downstream enzymes and their activities are cytoplasmic. We speculate that the PanKs may act as sensors of CoA homeostasis in the diverse locations, as PanK activity is modulated by metabolites such as acetyl-CoA, acyl-carnitine [10], and acylethanolamide [11]. Our data indicate a possible role for PanK1α and hPanK2 as sensors of CoA homeostasis in the nucleus. The reduced amount of nuclear hPanK2 during G2 phase of the cell cycle correlates with the time when chromatin becomes more structured and less active in preparation for mitosis (Fig. 12). Nuclear protein acetylation plays an important epigenetic role in regulating gene expression and the availability of acetyl-CoA is thought to regulate this process [42]. Support for a nuclear role for PanK comes from experiments in the *Drosophila* system that suggest PanK may have a role in maintaining DNA integrity [43]. The *fumble* mutation in *D. melanogaster*, later identified as a PanK, results in hypomorphic larvae that exhibit cell division defects and abnormal chromosome segregation in dividing neuroblasts.

The localization of hPanK2 in the nucleus raises the interesting question of whether mutations associated with PKAN disease may affect a nuclear function rather than, or in addition to, its proposed role in mitochondrial CoA homeostasis.

## Materials and Methods

### Cell Lines

HEK293, HeLa and SH-SY5Y human cell lines were obtained from the American Type Culture Collection (ATCC). HEK293 and HeLa cells were cultured at 37°C in 5% CO_2_ in Dulbecco’s modified Eagle’s medium (DMEM) supplemented with 2 mM glutamine, 10% fetal bovine serum (FBS), 50 U/ml penicillin and 50 U/ml streptomycin (Invitrogen). For live cell imaging, cells were seeded in 4-well chamber slides (Lab-Tek II-CC2 treated, Nunc) at low density and were grown to 70% confluence for approximately 2 days. Transfections were performed with 0.2–0.5 µg DNA/chamber using Lipofectamine 2000 (Invitrogen) according to the manufacturers’ recommendations. Cells were imaged 24 hours after transfection at approximately 70%–80% confluence. For imaging experiments, DMEM without phenol red supplemented with 15 mM HEPES pH 7.4 and OxyFluor (1∶100 dilution, Oxyrase, Inc) was utilized. When indicated, cycloheximide was added 45 minutes prior to imaging. SH-SY5Y cells were cultured at 37°C in 5% CO_2_ in Minimal Essential Medium/Ham’s F-12 with 1 mM glutamine, 10% FBS, 50 U/ml penicillin and 50 U/ml streptomycin. Stable HEK293 cell lines that expressed His-tagged mouse PanK1α or His-tagged human PanK1α were generated. Cells were either transfected with plasmid pPJ375 which encoded mouse PanK1α-His, or transduced with an amphotropic retrovirus expressing hPanK1α-His (pAA130), and subsequently grown in the presence of either 400 µg/ml of G418 or 3 µg/mL of puromycin, respectively, to select clones with genomic integration of the cDNAs. Single colonies were screened for expression of the His-tag by immunoblotting cell lysates following fractionation by SDS-PAGE. The anti-His-tag antibody was purchased from Santa Cruz Biotechnology. Positive cell clones were used for fixed cell imaging experiments.

### Cell Synchronization

HeLa cells were synchronized by arrest at the G1/S boundary using a double thymidine block as described [30]. Cells were grown to about 40% confluence and thymidine (Sigma) was added to a final concentration of 2 mM. Cells were incubated at 37°C for 16 hrs, washed with phosphate-buffered saline (PBS) three times, and incubated again for 8–12 hours in DMEM +10%FBS. A second treatment with 2 mM thymidine was performed and cells were incubated for another 16 hrs. Finally, the cells were washed three times with PBS and fresh media was added to release the cells from arrest and enable cell cycle progression of the synchronized population during the next 12 hrs.

### Cell Cycle Analysis by Flow Cytometry

Synchronized HeLa cells were trypsinized and washed in PBS twice, fixed in 70% ethanol, and stained with propidium iodide (50 µg/ml) in the presence of RNase (100 µg/mL) in a sodium citrate buffer (40 mM). Stained cells were washed and diluted in PBS and passed through the BD FACS-Calibur flow system (BD Biosciences). Data were appropriately gated and analyzed by the Cell Quest Pro software.

### Digitonin Permeabilization and Immunobloting

Experiments were performed following previously described methodology [32], [44], [45]. SH-SY5Y cells at 80% confluence were incubated with 0.002–8.0 mg/ml digitonin in mitochondrial isolation buffer for 15 min. at 37°C. Mitochondrial isolation buffer consisted of 215 mM mannitol, 75 mM sucrose, 20 mM Hepes, 1 mM EGTA, and 1X complete protease inhibitor (Roche), pH 7.2. Complete solubilization of cell membranes was accomplished by addition of 0.1% Triton X-100. Following detergent treatment, the cells were centrifuged at 22,800×*g* for 10 min. The supernatant proteins were fractionated by SDS-PAGE and identified by immunoblotting. Sources of antibodies were: second mitochondria-derived activator of caspases SMAC (Cat. #61225, BD Transductions Laboratories), Cyclophilin D (Cat. #MSA04 Mitosciences). The antibody that recognized hPanK2 protein was described previously [5]. For Proteinase K treatment, the protease inhibitors were not added to the mitochondrial isolation buffer and the pellets obtained from the digitonin lysates were resuspended to a concentration of 2–4 mg/ml in the mitochondrial isolation buffer and incubated with proteinase K (50 µg/ml) for 10 min. at 37°C. Phenylmethylsulfonyl fluoride was added to a final concentration of 2 mM to inhibit the Proteinase K activity followed by further incubation at 37°C. Lysates were centrifuged at 13,000×*g* for 10 min. and pellets were fractionated by SDS-PAGE followed by immunoblotting.

### Molecular Constructs

For imaging the human PanK proteins, the plasmid pAA130, containing the full length hPanK1α fused to a His-tag, was generated by PCR amplification from plasmid pPJ350 using *Pfx* polymerase (Invitrogen) and primers containing AgeI and EcoRI restriction sites for directional cloning (Table S2). A 1.8 kb PCR product was purified (Gel Extraction Kit, Qiagen) and ligated into pCR-Blunt II-TOPO cloning vector (Invitrogen). The plasmid was digested with AgeI and EcoRI (Promega) and the fragment subcloned into retroviral bicistronic QCXIP vector (Clontech). To generate plasmids pAA132 and pAA133 encoding the truncated mutants of hPanK1α (amino acids 1–235 and 1–217) fused to the ZsGreen1 fluorescent protein in the ZsGreen1-N1 expression vector (Clontech), the same techniques were used with primers including NheI and HindIII restriction sites. The plasmid pAA134 containing amino acids 218–233 of hPanK1α fused to ZsGreen1 was obtained by insertion of two complementary oligonucleotides encoding a Kozak sequence (GTC GCC ACC), an ATG starting codon, the desired region of hPanK1α and NheI or HindIII restriction hemisites at each 5′- and 3′-end, respectively (Table S2).

The Plasmid pAA121, encoding hPanK1β fused to ZsGreen1, was generated by PCR from pGR20 plasmid and using primers containing EcoRI and AgeI restriction sites (Table S2). The plasmid pAA124 encoding hPanK3 fused to ZsGreen1 was generated by PCR amplification from pCMV-SPORT6-hPanK3 (Open Biosystems, ID: #4343270) using primers containing EcoRI and AgeI restriction sites (Table S2).

For imaging the full length hPanK2 protein, the ORF sequence was obtained from pKM4 plasmid. After removal of the stop codon and generation of an EcoRI restriction site by site-directed mutagenesis (QuikChange Lightning Site-directed Mutagenesis Kit, Agilent Technologies), a plasmid pAA273 was generated. This plasmid was digested using NheI and EcoRI restriction enzymes and a 1.7 kb insert (corresponding to the hPanK2 ORF without the stop codon) was ligated into the ZsGreen1-N1 vector, generating plasmid pAA309 (Table S3). To generate plasmids encoding the truncated mutants of hPanK2: pAA321 (amino acids 82–570), pAA241 (amino acids 1–210), pAA197 (amino acids 1–150), pAA249 (amino acids 82–210) and pAA308 (amino acids 1–52), the same techniques were used with indicated primers containing restriction sites for directional cloning into ZsGreen1-N1 vector (Table S3). The plasmid pAA251 (amino acids 82–94) as well as the plasmid pAA303 (amino acids 268–275) of hPanK2 were obtained by insertion of two complementary oligonucleotides encoding a Kozak sequence (GTC GCC ACC), an ATG inititation codon, the desired region of hPanK2 and NheI or HindIII restriction hemisites at each 5′- and 3′-end, respectively (Table S3).

For imaging the mouse PanK proteins, the plasmid pPJ352 encoding the full length mouse PanK1α fused to a His-tag was generated by PCR amplification of the N-terminal region including residues 1 to 363 of mouse PanK1α using the plasmid pPanK1α as a template. The forward primer introduced an AflII restriction site and the reverse primer included the internal HpaI restriction site and a His-tag (Table S4). The PCR product was first ligated into pCR2.1 (Invitrogen), then digested with AflII and HpaI, and the insert used to substitute the tag-less AflII-HpaI portion of the *Pank1α* gene contained in the pPanK1a expression vector (Invitrogen). The plasmids pRL015, pRL017, pRL019, pAA143 and pAA145, encoding truncated mutants of mouse PanK1α (amino acids 1–185, 1–60, 61–185, 9–185 and 9–150 respectively), were obtained by PCR from pPanK1 plasmid as a template, and the corresponding PCR products were ligated into pCR-Blunt II-TOPO vector (Invitrogen) and finally subcloned into ZsGreen1-N1 vector using NheI and HindIII restriction enzymes (Table S4). Similarly, the plasmid pAA144 encoding amino acids 168–185 of mouse PanK1α fused to ZsGreen1 was obtained using plasmid pRL019 as a template, and a XhoI restriction site from pCR-Blunt II-TOPO vector was utilized at the 5′-end together with HindIII for subcloning into pZsGreen1-N1 vector (Table S4). The plasmid pAA146 encoding amino acids 61–150 of mouse PanK1α fused to ZsGreen1 originated from pAA145. The plasmid pAA150 was obtained by insertion of two complementary oligonucleotides encoding a Kozak sequence (GTC GCC ACC), the first 8 amino acids of mouse PanK1α and NheI and HindIII hemisites at the 5′- and 3′- ends, respectively (Table S4).

Plasmid pAA126 encoding the full length mouse PanK1β fused to ZsGreen1-N1 was generated by PCR amplification from pRC63 plasmid and primers containing EcoRI restriction sites (Table S4). Plasmid pAA3388 encoding the full length mouse PanK2 in the ZsGreen1-N1 vector was generated by PCR from pPJ256 as a template using specific primers containing NheI and HindIII restriction sites (Table S4). Plasmid pAA128 encoding the full length mouse PanK3 in the ZsGreen1-N1 vector was generated by PCR from pPJ218 as a template using specific primers containing EcoRI and AgeI restriction sites (Table S4). Amino acid substitutions performed in the human and/or mouse PanK1α and hPanK2 proteins were introduced in sequential steps by site-directed mutagenesis using QuickChange XL mutagenesis kit (Agilent Technologies). The complete list of oligonucleotides is shown in Table S5.

For imaging the nuclear membrane, the plasmid mCherry-Lmn A/C was generated by replacing the sequence of fluorescent CFP from CFP-Lamin A/C construct (gift from Dr. Vicente Andres, Instituto de Biomedicina de Valencia) for the fluorescent mCherry-C1 sequence (Clontech) using NheI and BglII restriction enzymes (Promega). For imaging chromatin, the plasmid pAA086 encoding human heterochromatin protein 1 alpha (hHP1a) fused to mCherry was obtained by transferring the hHP1a insert from pcDNA3.0-Venus-HP1a (gift from Dr. Inoue Akira, St. Jude Children’s Research Hospital) using EcoRI and HpaI restriction enzymes within the mCherry-C1 vector. For imaging the nucleolus, the plasmids pAA075 and pAA076 encoding the ORF sequence of hFibrillarin fused to ZsGreen1 or mCherry fluorescent proteins, respectively, were generated by PCR amplification from plasmid pOTB7-hFibrillarin as a template (Open Biosystems, clone ID: #3504198) with primers containing EcoRI and AgeI restriction sites (Table S1). A 1.0 kb PCR product was purified from agarose gel using Gel Extraction Kit (Qiagen) and ligated into pCR-Blunt II-TOPO cloning vector (Invitrogen). The resulting plasmid pAA074 was digested with EcoRI and AgeI restriction enzymes (Promega) and the insert was subcloned into ZsGreen1-N1 and mCherry-N1 expression plasmids. The plasmid pAA079 encoding hB23 fused to mCherry was obtained by PCR from template pCMV-SPORT6-hB23 plasmid (Open Biosystems, clon ID: #3877633) and using primers containing EcoRI and BamHI restriction sites (Table S1).

For imaging peroxisomes, the plasmid pAA084 encoding mCherry fused to a peroxisomal targeting sequence from firefly luciferase (GenBank: AAC53658.1) was generated by the insertion of two complementary oligonucleotides containing BglII and EcoRI hemisites (Table S1). For imaging Lysosomes, the plasmid Lamp1-mCherry was obtained from Samuel Connell (St. Jude Children’s Research Hospital). For imaging endosomes, the plasmids pAA105 and pAA107 encoding human Rab5 and Rab11 fused to mCherry were obtained by replacing GFP sequence from Rab5-GFP and Rab11-GFP plasmids (kindly supplied by Dr. Guillermo Gomez, Universidad Nacional de Cordoba) using AgeI and BgIII restriction enzymes. To image clathrin-associated vesicles, the plasmid pAA209 encoding human Clathrin LCB fused to mCherry was generated by PCR from plasmid pOTB7-Clathrin LCB as a template (Open Biosystems, clone ID: 4299637) with primers containing a XhoI and BamHI restriction sites (Table S1). A 0.65 kb PCR product was purified and ligated into pCR-Blunt II-TOPO cloning vector (Invitrogen). The resulting plasmid (pAA181) was digested with the same enzymes and the insert subcloned into mCherry-C1 expression plasmid. Finally, the whole construct was released by digestion using AgeI and BamHI and subcloned into QCXIP retroviral expression plasmid for retroviral production. All constructs were verified by DNA sequencing (Hartwell center for Biotechnology at St. Jude Children’s Research Hospital).

### Immunostaining

For imaging of PanK1α isoforms, HEK293 with stable expression of pPJ375 (mPanK1α-His) or pAA130 (hPanK1α-His) were grown on 4-chamber slides (Lab-Tek™). Slides were fixed with ice cold methanol for 20 minutes at −20°C and blocked with 3% BSA in PBS for 1 h at 25°C and then incubated for 1 hour at 25°C with PBS-1% BSA buffer containing diluted (1/250) rabbit primary anti-His-Tag polyclonal antibody (cat. #2365, Cell Signaling), followed by 1 hour incubation with Alexa Fluor 488 goat anti-rabbit IgG antibody (1/1000, Invitrogen) at 25°C. Cells were washed 3 times during 10 minutes with PBS and finally rinsed with ultrapure water to remove excess of salts. Finally, cells were mounted using Prolong® Gold antifade reagent with DAPI (Molecular Probes). For imaging of hPanK2 during cell cycle progression, HeLa cells were immunostained as described above, using the anti-hPanK2 specific antibody described previously [5].

### Live-cell Confocal Imaging

For live cell imaging experiments, cells were transfected with PanK fusión constructs, for approximately 24 hours, and approximately 30 minutes before utilization where indicated, nuclei, plasma membrane and/or mitochondria were counterstained with Hoechst 33342 (5 µg/ml), with Alexa Fluor® 647 wheat germ agglutinin conjugate (Molecular Probes), and/or Mitotracker Red CMXRos (Cat, no M7512, Invitrogen), respectively. All images were acquired using an Nikon C1si inverted laser scanning confocal microscope (60X apochromatic, 1,45 NA objective), and images were assessed using the EZ-C1 3.20 Viewer (Nikon Corporation), exported as TIFF files, deconvoluted using Image J and figures were assembled using Adobe Photoshop CS4 software. For photoswitching experiments with hPanK2-Dendra2, a 3i Marianas SDC inverted digital microscope was utilized (Intelligent Imaging Innovations/3i, Denver, CO). This workstation consists of a CSU22 confocal head (Yokogowa Electric Corporation), DPSS lasers (CrystaLaser), and a Carl Zeiss 200M motorized inverted microscope (Carl Zeiss MicroImaging, Thornwood), equipped with spherical aberration correction optics. Temperature was maintained at 37°C and CO_2_ at 5% v/v in a humidified atmosphere using an environmental control chamber (Solent Scientific). Images were acquired with a Zeiss Plan-Neofluar 40× 1.3 NA DIC objective on a Cascade II 512 EMCCD (Photometrics, Tucson, AZ), using SlideBook 4.2 software (3i).

### Sequence Analysis

Alignments were made using the SMS tool from “The Sequence Manipulation Suite: JavaScript programs for analyzing and formatting protein and DNA sequences” [46]. For nuclear exportation signal prediction, NetNES 1.1 server was used [25].

## Supporting Information

Figure S1 **Deletion of catalytic domain leads to accumulation of hPanK2(1–210)-Zsgreen1 fusion protein in the nucleus.** (A) Schematic diagram of the hPanK2(1–570) and hPanK2(1–210) fused to ZsGreen1 protein. Numbers indicate hPanK2 amino acid positions. The mitochondrial targeting signal (MTS, yellow), nuclear localization signal (NLS, cyan) and nuclear export signal (NES, orange) are indicated. (B) HeLa cells were transfected with either construct and visualized using live-cell confocal microscopy. (C) HeLa cells were scored for subcellular distribution of hPanK2(1–570)-ZsGreen1 (n = 526) and hPanK2(1–210)-ZsGreen1 (n = 365) as [nuclear and mitochondrial] (gray bars) or mitochondrial only (black bars). Significance was determined using unpaired Students t-test. ***p<0.001. Scale bar, 10 um.
(TIF)

Figure S2 **Human PanK2 has a functional NES.** Scoring of transfected HeLa cells (hPanK2(268–275)-ZsGreen1, n = 129; ZsGreen1, n = 155) for subcellular distribution of hPanK2(268–275)-ZsGreen1 as primarily nuclear “N”, both nuclear and cytoplasmic “NC” or primarily cytoplasmic “C”. Transfected cells were classified based on the overlapping fluorescence patterns illustrated in Figure 8C, panels c and f.
(TIF)

Figure S3 **Leptomycin B treatment leads to accumulation of the hPanK2(82–570)-mCherry fusion protein in the nucleus.** (A) HeLa cells were transiently transfected with the expression plasmid pAA283 encoding hPanK2(82–570) fused to mCherry (without MTS) and after 24 hours cells were treated with cycloheximide (50 µg/ml) with or without Leptomycin B (LMB) (20 nM) as indicated and visualized by live-cell confocal imaging 2.5 hours after treatment. Dashed lines delimit cell borders. Cells were scored according to the fluorescence distribution as nuclear only “N” or both nuclear and cytoplasmic “NC”. (+ LMB, n = 308; Untreated, n = 407). Significance was determined using unpaired Students t-test. ***p<0.001. Scale bar, 10 µm. (C and D) Hela cells were seeded in 6 well plates and the following day they were treated with cycloheximide (50 µg/ml) and LMB (20 nM) and total adherent cell number and % viability were determined hourly up to 8 hours. Cells were counted in triplicate utilizing an automatic counter (Nucleocounter, Chemometec).
(TIF)

Figure S4 **Quantification of nuclear hPanK2 during the cell cycle.** HeLa cells were arrested at the G1/S phase boundary of the cell cycle using a double thymidine block as described in Materials and Methods. Following release from the block, the synchronized cells progressed through the cell cycle and at the specificed times, cells were fixed and immunostained to localize the endogenous hPanK2 protein. The DAPI-stained compartment in interphase cells was defined as the nucleus and the amount of fluorescent nuclear hPanK2 was quantified in arbitrary units (a.u.) using image analysis software. All images were obtained from single optical slices and the microscopic settings were the same for all images. Data are presented as the mean ± s.e. of more than 100 cells per group. Significance of the data relative to arrested cells at the G1/S boundary (T = 0) was determined using unpaired Students t-test. *p<0.05; ****p<0.0001.
(TIF)

Table S1 **Plasmids and Primers.** Restriction site sequences are underlined.
(DOCX)

Table S2 **Human PanK1 and PanK3 Plasmids and Primers.** hPanK1α sequence was inserted in retroviral bicistronic expression QCXIP vector (Clontech). The other hPanK inserts were subcloned in the fluorescent vector ZsGreen1-N1 (Clontech). Restriction site sequences are underlined.
(DOCX)

Table S3 **Human PanK2 Plasmids and Primers.** All hPanK constructers were inserted in the fluorescent vector ZsGreen1-N1 (Clontech). hPanK2(1–570) was also inserted in the photoactivatable Dendra2-N1 vector (Clontech), named pAA275. Restriction site sequences are underlined.
(DOCX)

Table S4 **Mouse PanK1 and PanK3 Plasmids and Primers.** mPanK1α sequence was inserted in pcDNA3.1+ expression vector (Invitrogen). The other mPanK inserts were inserted in the fluorescent vector ZsGreen1-N1 (Clontech). Restriction site sequences are underlined.
(DOCX)

Table S5 **Site-Directed Mutagenesis Plasmids and Primers.** hPanK1α-noNLS-His was made using pAA130 as template. mPanK1α-noNLS-His was made using pPJ352 as template. hPanK2(82-570-noNLS)-mCherry and hPanK2(82-570-noNES)-mCherry were made using pAA283 as template.
(DOCX)
