# Supplementary figures and images for: Compartmentalization of Mammalian Pantothenate Kinases
Source: PLoS One. 2012 Nov 13;7(11):e49509. doi: 10.1371/journal.pone.0049509 (PMC3496714; doi:10.1371/journal.pone.0049509)

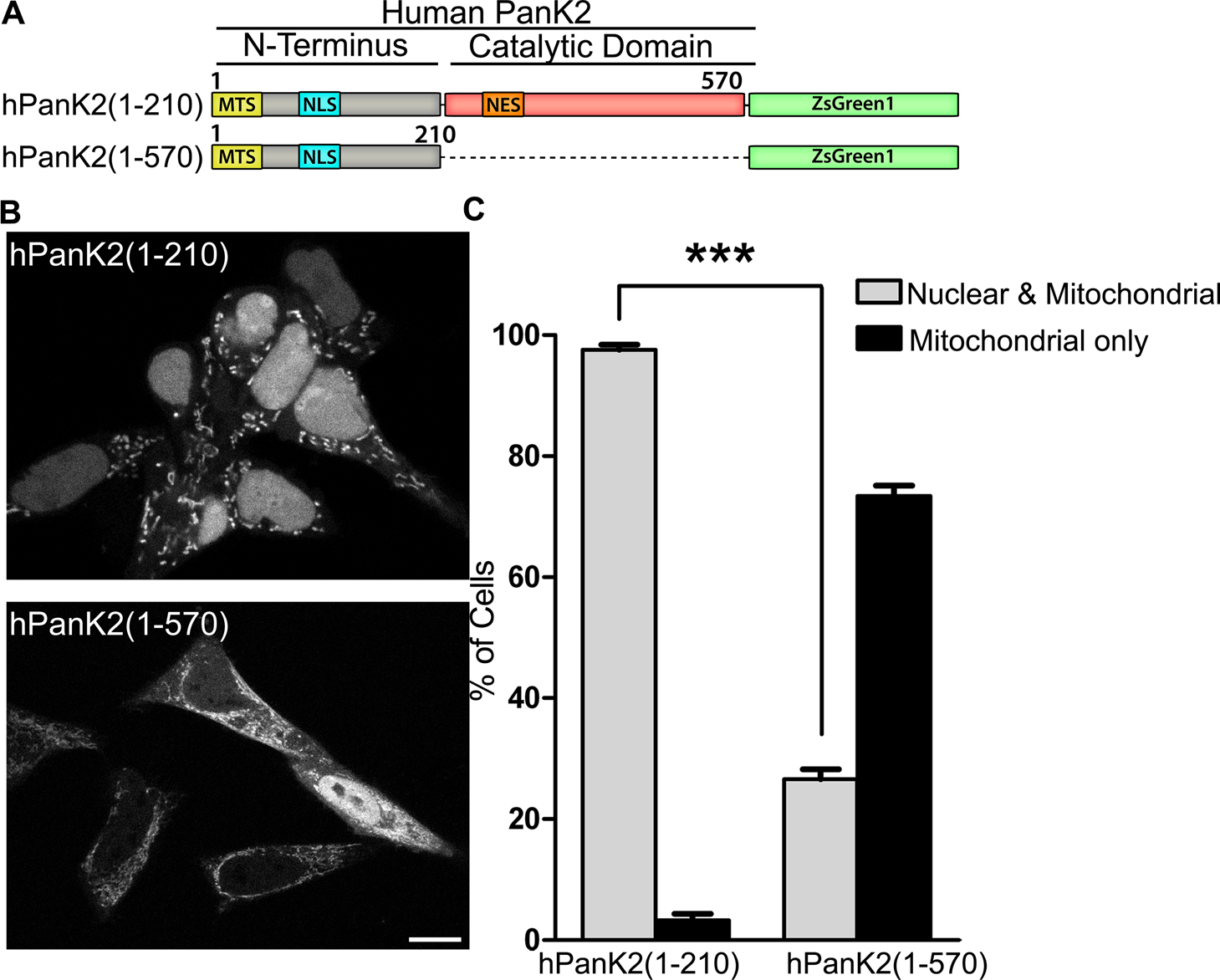

Supplement: Figure S1 — Deletion of catalytic domain leads to accumulation of hPanK2(1–210)-Zsgreen1 fusion protein in the nucleus. (A) Schematic diagram of the hPanK2(1–570) and hPanK2(1–210) fused to ZsGreen1 protein. Numbers indicate hPanK2 amino acid positions. The mitochondrial targeting signal (MTS, yellow), nuclear localization signal (NLS, cyan) and nuclear export signal (NES, orange) are indicated. (B) HeLa cells were transfected with either construct and visualized using live-cell confocal microscopy. (C) HeLa cells were scored for subcellular distribution of hPanK2(1–570)-ZsGreen1 (n = 526) and hPanK2(1–210)-ZsGreen1 (n = 365) as [nuclear and mitochondrial] (gray bars) or mitochondrial only (black bars). Significance was determined using unpaired Students t-test. ***p<0.001. Scale bar, 10 um. (TIF) [file pone.0049509.s001.tif]

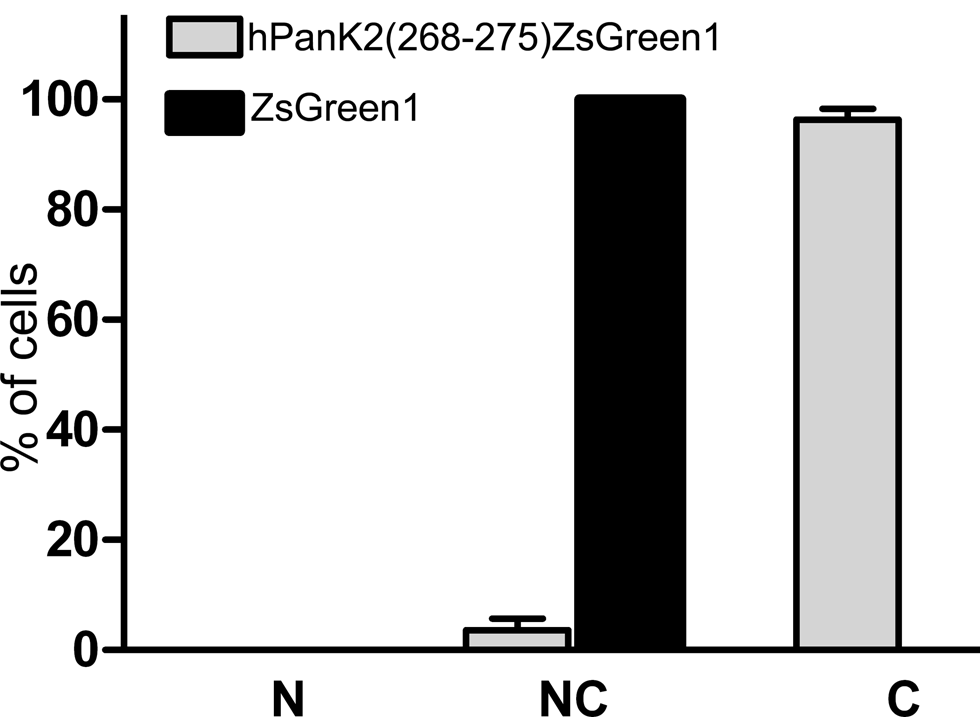

Supplement: Figure S2 — Human PanK2 has a functional NES. Scoring of transfected HeLa cells (hPanK2(268–275)-ZsGreen1, n = 129; ZsGreen1, n = 155) for subcellular distribution of hPanK2(268–275)-ZsGreen1 as primarily nuclear “N”, both nuclear and cytoplasmic “NC” or primarily cytoplasmic “C”. Transfected cells were classified based on the overlapping fluorescence patterns illustrated in Figure 8C, panels c and f. (TIF) [file pone.0049509.s002.tif]

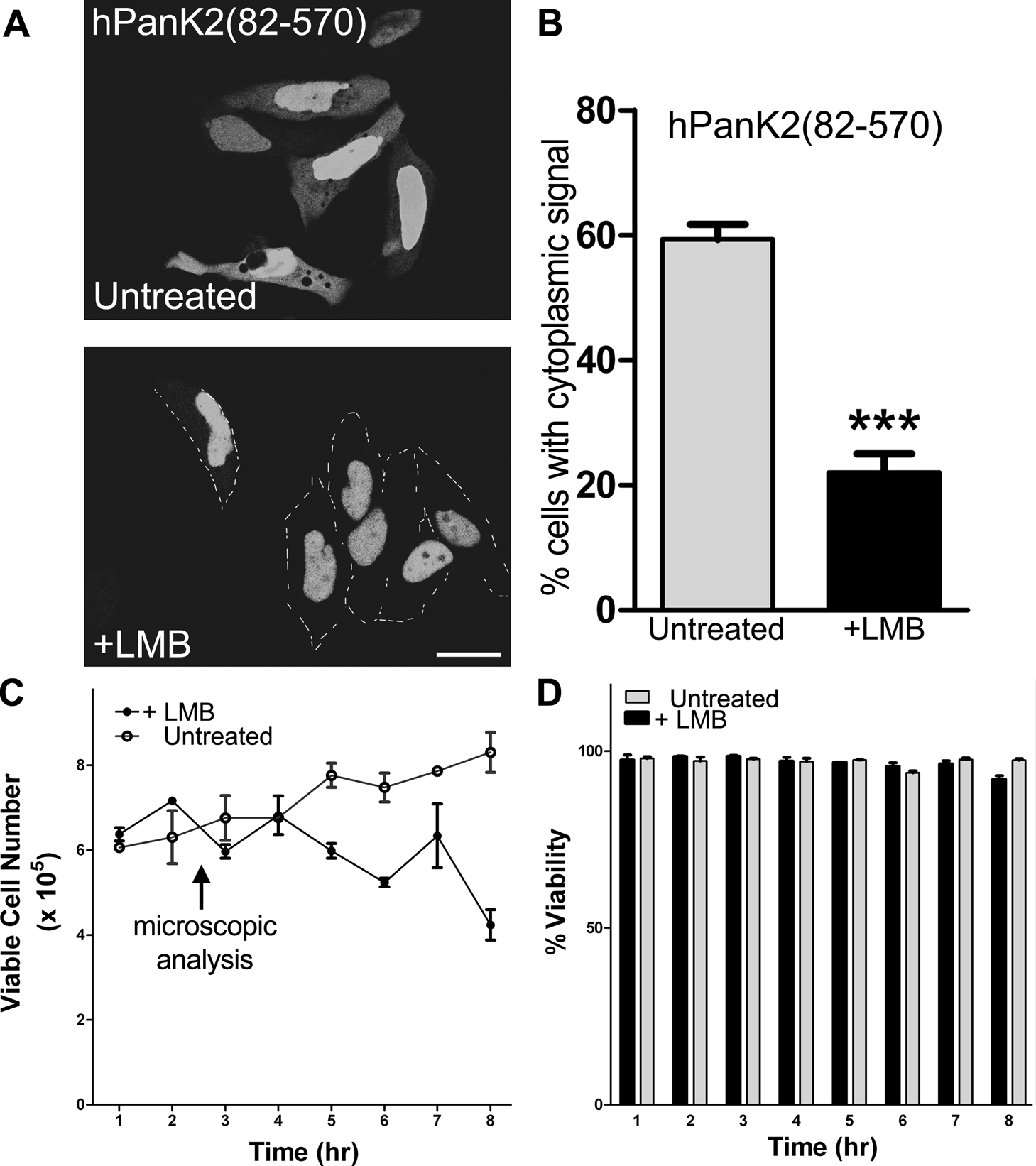

Supplement: Figure S3 — Leptomycin B treatment leads to accumulation of the hPanK2(82–570)-mCherry fusion protein in the nucleus. (A) HeLa cells were transiently transfected with the expression plasmid pAA283 encoding hPanK2(82–570) fused to mCherry (without MTS) and after 24 hours cells were treated with cycloheximide (50 µg/ml) with or without Leptomycin B (LMB) (20 nM) as indicated and visualized by live-cell confocal imaging 2.5 hours after treatment. Dashed lines delimit cell borders. Cells were scored according to the fluorescence distribution as nuclear only “N” or both nuclear and cytoplasmic “NC”. (+ LMB, n = 308; Untreated, n = 407). Significance was determined using unpaired Students t-test. ***p<0.001. Scale bar, 10 µm. (C and D) Hela cells were seeded in 6 well plates and the following day they were treated with cycloheximide (50 µg/ml) and LMB (20 nM) and total adherent cell number and % viability were determined hourly up to 8 hours. Cells were counted in triplicate utilizing an automatic counter (Nucleocounter, Chemometec). (TIF) [file pone.0049509.s003.tif]

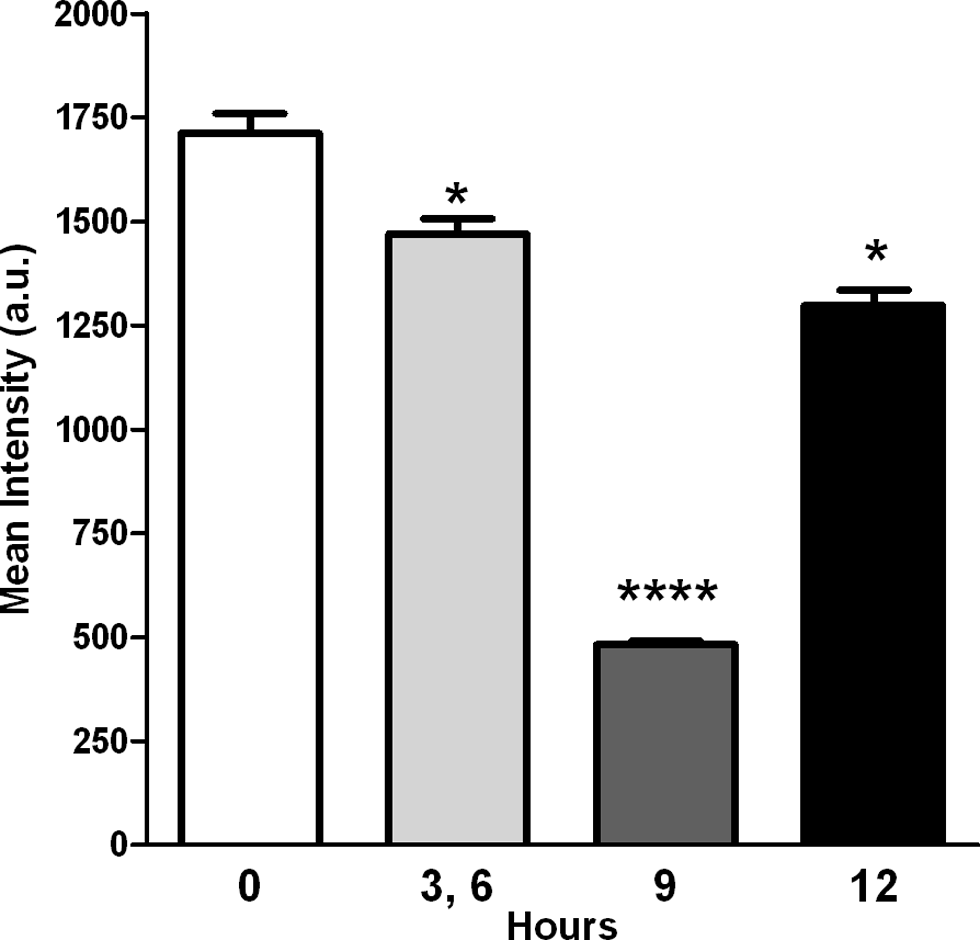

Supplement: Figure S4 — Quantification of nuclear hPanK2 during the cell cycle. HeLa cells were arrested at the G1/S phase boundary of the cell cycle using a double thymidine block as described in Materials and Methods. Following release from the block, the synchronized cells progressed through the cell cycle and at the specificed times, cells were fixed and immunostained to localize the endogenous hPanK2 protein. The DAPI-stained compartment in interphase cells was defined as the nucleus and the amount of fluorescent nuclear hPanK2 was quantified in arbitrary units (a.u.) using image analysis software. All images were obtained from single optical slices and the microscopic settings were the same for all images. Data are presented as the mean ± s.e. of more than 100 cells per group. Significance of the data relative to arrested cells at the G1/S boundary (T = 0) was determined using unpaired Students t-test. *p<0.05; ****p<0.0001. (TIF) [file pone.0049509.s004.tif]
