# Supplementary material for: Compartmentalization of Mammalian Pantothenate Kinases
Source: PLoS One. 2012 Nov 13;7(11):e49509. doi: 10.1371/journal.pone.0049509 (PMC3496714; doi:10.1371/journal.pone.0049509)
Supplement: Table S1 — Plasmids and Primers. Restriction site sequences are underlined. (DOCX) [file pone.0049509.s005.docx]

| **Table S1.** | | | | | |  |
| --- | --- | --- | --- | --- | --- | --- |
| **Name** | **Plasmid** | | **Primer** | **Sequence (5’→3’)** |  |  |
| hFibrillarin | pAA075/ pAA076 | | hFib-EcoRI-for | GAATTCTCGCCACCATGAAGCCAGGATTCAGT |  |  |
|  | | | hFib-AgeI-rev | ACCGGTGCGTTCTTCACCTTGGGGGGTGGCCTGTAC |  |  |
| hB23 | pAA079 | | hB23-EcoRI-for | GAATTCGATGGAAGATTCGATGGACATGGAC |  |  |
|  | | | hB23-BamHI-rev | GGATCCTTAAAGAGACTTCCTCCACTGCCAGAG |  |  |
| SKL | pAA084 | | PTS-BglII-for | GATCTAGAGAGATCCTCATAAAGGCCAAGAAGGGCGGAAAGTCCAAATTGTAAG |  |  |
|  | | | PTS-EcoRI-rev | AATTCTTACAATTTGGACTTTCCGCCCTTCTTGGCCTTTATGAGGATCTCTCTA |  |  |
| Clathrin LCB | pAA209 | | Clathrin LCB-XhoI-f | CTCGAGGGATGGCTGATGACTTTGGCTTCTTC |  |  |
|  | | Clathrin LCB-BamHI-rev | | GGATCCTAGCGGGACAGTGGCGTCTGCTTC | |  |
|  | | | | | | |
