# Supplementary material for: Compartmentalization of Mammalian Pantothenate Kinases
Source: PLoS One. 2012 Nov 13;7(11):e49509. doi: 10.1371/journal.pone.0049509 (PMC3496714; doi:10.1371/journal.pone.0049509)
Supplement: Table S2 — Human PanK1 and PanK3 Plasmids and Primers. hPanK1α sequence was inserted in retroviral bicistronic expression QCXIP vector (Clontech). The other hPanK inserts were subcloned in the fluorescent vector ZsGreen1-N1 (Clontech). Restriction site sequences are underlined. (DOCX) [file pone.0049509.s006.docx]

| **Table S2.** | | | |
| --- | --- | --- | --- |
| **Name** | **Plasmid** | **Primer** | **Sequence (5’→3’)** |
| hPanK1α^a^ | pAA130 | hPanK1α-AgeI-for | ACCGGTCGCCACCATGTTGAAACTCGTCGGTGGCGGT |
|  | | hPanK1α-His-rev | TCAATGATGATGATGATGATGCTTGTCATCAGTCATTTTGAACAGTTCCAAC |
| hPanK1α(1-235) | pAA132 | hPanK1α-NheI-for | GCTAGCCCATGTTGAAACTCGTCGGTGGCGGT |
|  | | hPanK1α-235-HindIII-rev | AAGCTTTGGCGGCCTGTTCTTTCTCCCCGAG |
| hPanK1α(1-217) | pAA133 | hPanK1α-NheI-for | (See pAA132) |
|  | | hPanK1α-217-HindIII-rev | AAGCTTGGCCGGGGAGTCATGCTGAGGGAGC |
| hPanK1α(218-233) | pAA134 | hPanK1α-(218-233)-for | CTAGCGTCGCCACCATGAAGAAATGCCGGCTGCGGAGGAGGATGGACTCGGGGAGAAAGAACAGGA |
|  | | hPanK1α-(218-233)-rev | AGCTTCCTGTTCTTTCTCCCCGAGTCCATCCTCCTCCGCAGCCGGCATTTCTTCATGGTGGCGACG |
| hPanK1β | pAA121 | hPanK1β-EcoRI -for | GAATTCAGTCGCCACCATGAAGCTTATAAATGGCAAAAAGC |
|  | | hPanK1-AgeI-rev | ACCGGTGCCTTGTCATCAGTCATTTTGAACAGTTCCAAC |
| hPanK3 | pAA124 | hmPanK3-EcoRI-for | GAATTCAGTCGCCACCATGAAGATCAAGGATGCCAAGAAACCCTC |
|  | | hmPanK3-AgeI-rev | ACCGGTGCGCTGAAATTTGGCAGTCCAAGAAGTGCACCAAC |
|  | | | |
