# Supplementary material for: Compartmentalization of Mammalian Pantothenate Kinases
Source: PLoS One. 2012 Nov 13;7(11):e49509. doi: 10.1371/journal.pone.0049509 (PMC3496714; doi:10.1371/journal.pone.0049509)
Supplement: Table S3 — Human PanK2 Plasmids and Primers. All hPanK constructers were inserted in the fluorescent vector ZsGreen1-N1 (Clontech). hPanK2(1–570) was also inserted in the photoactivatable Dendra2-N1 vector (Clontech), named pAA275. Restriction site sequences are underlined. (DOCX) [file pone.0049509.s007.docx]

| **Table S3.** | | | |
| --- | --- | --- | --- |
| **Name** | **Plasmid** | **Primer** | **Sequence (5’→3’)** |
| hPanK2(1-570) | pAA309 | hPanK2-SDM-EcoRI-3-for | GTTGAAGATCCCGGGAATTCATTACCTGGGG |
|  | | hPanK2-SDM-EcoRI-3-rev | CCCCAGGTAATGAATTCCCGGGATCTTCAAC |
| hPanK2(1-210) | pAA241 | hPanK2-Bglll-for | AGATCTGCCACCATGAGGAGGCTCGGGCCCTTCCACCCA |
|  | | hPanK2-EcoRI-210-rev | GAATTCCAAGCGGCCGCTTTTTCCTCAGGCTTTCGACGCGCT |
| hPanK2(1-150) | pAA197 | hPanK2-Bglll-for | (See pAA241) |
|  | | hPanK2-EcoRI-150rev | GAATTCCCTGCTCCCCAGCCGACGAGACGGAGGTGGCGG |
| hPanK2(95-570) | pAA359 | hPanK2-Nhel-for | GCTAGCATGCTCTGCTCTGGCTGGACT |
|  | | hPanK2-EcoRI-rev | TACCGTCGACTGCAGAATTCCCGGGAT |
| hPanK2(1-52) | pAA308 | hPanK2-Bglll-for | (See pAA241) |
|  | | hPanK2-52-EcoRI-rev | TATTGAATTCCGTCCAATGAGAGGCTATCGT |
| hPanK2(82-570) | pAA321 | hPanK2-82-for | ATGGCTATGCGTTGGCGCAACGGAAGA |
|  | | hPanK2-SDM-EcoRI-3-rev | (see pAA309) |
| hPanK2(82-210) | pAA249 | hPanK2-82-For | ATGGCTATGCGTTGGCGCAACGGAAGA |
|  | | hPanK2-EcoRI-210-rev | (See pAA241) |
| hPanK2(82-94) | pAA251 | hPanK2(82-94)-f | CTAGCGTCGCCACCATGAGATGGAGAAATGGAAGAGGAGGACGTCCAAGAGCTCGAA |
|  | | hPanK2(82-94)-rev | AGCTTTCGAGCTCTTGGACGTCCTCCTCTTCCATTTCTCCATCTCATGGTGGCGACG |
| hPanK2(268-275) | pAA303 | hPanK2(268-275)-for | CTAGCGTCGCCACCATGCTCGAGCTGAAGGACCTGACTCTGGAA |
|  | | hPanK2(268-275)-rev | CCGGTTCCAGAGTCAGGTCCTTCAGCTCGAGCATGGTGGCGACG |
|  | | | |
