# Supplementary material for: Compartmentalization of Mammalian Pantothenate Kinases
Source: PLoS One. 2012 Nov 13;7(11):e49509. doi: 10.1371/journal.pone.0049509 (PMC3496714; doi:10.1371/journal.pone.0049509)
Supplement: Table S4 — Mouse PanK1 and PanK3 Plasmids and Primers. mPanK1α sequence was inserted in pcDNA3.1+ expression vector (Invitrogen). The other mPanK inserts were inserted in the fluorescent vector ZsGreen1-N1 (Clontech). Restriction site sequences are underlined. (DOCX) [file pone.0049509.s008.docx]

| **Table S4.** | | | | | |  |
| --- | --- | --- | --- | --- | --- | --- |
| **Name** | **Plasmid** | | **Primer** | | **Sequence (5’→3’)** |  |
| mPanK1α ^a^ | pPJ352 | | mPanK1α-363-HpaI-for | | CCCTATGTTGCTGGTTAACATG |  |
|  | | | mPanK1α-548-His-XhoI-rev | | CTCGAGCTAGTGGTGGTGGTGGTGGTGCTGTGTATCGGTCATTTTGAACAGTTCCAACAGGGCCCC |  |
| mPanK1α(1-185) | pRL015 | | mPanK1α-1-NheI-for | | GCTAGCATGAGGGGGAGGCGGGCTCC |  |
|  | | | mPanK1α-185-HindIII-rev | | AAGCTTTGGCGGCCTGTTCTTCCTCCC |  |
| mPanK1α(9-185) | pAA143 | | mPank1α-9-NheI-for | | ATTGCGCTAGCATGCCGGGGTCCACCGAGGTTCCCGCAGC |  |
|  | | | mPanK1α-185-HindIII-rev | | (See pRL015) |  |
| mPanK1α(61-185) | pRL019 | | mPanK1α-61-NheI-for | | GCTAGCATGCCGAGGCTGCGGGAGAGCAAGCCCC |  |
|  | | | mPanK1α-185-HindIII-rev | | (See pRL015) |  |
| mPanK1α(168-185) | pAA144 | | mPank1α-168-for | | GTCGCCACCATGAAGAAATGCCGGCTGCGGAGGAGG |  |
|  | | | ZsGreen1-N1-rev | | TCACGCCGTAGAACTTGGAC |  |
| mPanK1α(9-150) | pAA145 | | mPank1α-9-NheI-for | | (See pAA143) |  |
|  | | | | mPanK1α-150-HindIII-rev | AAGCTTGAGCTGGAAGCGTGGGGCCG | |
| mPanK1α(61-150) | | pAA146 | | mPanK1α-61-NheI-for | (See pRL019) | |
|  | | | | ZsGreen1-N1-rev | (See pAA144) | |
| mPanK1α(1-60) | | pRL017 | | mPanK1α-1-NheI-for | (See pRL015) | |
|  | |  | | mPanK1α-60-HindIII-rev | AAGCTTCGCCGCGGTCGGGAGCTGGT | |
| mPanK1α(1-8) | | pAA150 | | mPanK1α-(1-8)-for | CTAGCGTCGCCACCATGAGGGGGAGGCGGGCTCCCCGGA | |
|  | | | | mPanK1α-(1-8)-rev | AGCTTCCGGGGAGCCCGCCTCCCCCTCATGGTGGCGACG | |
| mPanK1β | | pAA126 | | mPanK1β-EcoRI-for | GAATTCAGTCGCCACCATGAAGCTTGTAAATGGCAGAAAGCAAAC | |
|  | | | | mPanK1-EcoRI-rev | AGAATTCCCTGTGTATCGGTCATTTTGAAC | |
| mPanK2 | | pAA338 | | mPanK2-NheI-for | GCTAGCCACCATGGGAGCGGGCCGGTTTGGCGCGCCT | |
|  | | | | mPanK2-HindIII-rev | AAGCTTGGGTATCTTCAACAGCTCGAGGAGTGCTCCA | |
| mPanK3 | | pAA128 | | hmPanK3-EcoRI-for | (See pAA124) | |
|  | | | | hmPanK3-AgeI-rev | (See pAA124) | |
