# Supplementary material for: Compartmentalization of Mammalian Pantothenate Kinases
Source: PLoS One. 2012 Nov 13;7(11):e49509. doi: 10.1371/journal.pone.0049509 (PMC3496714; doi:10.1371/journal.pone.0049509)
Supplement: Table S5 — Site-Directed Mutagenesis Plasmids and Primers. hPanK1α-noNLS-His was made using pAA130 as template. mPanK1α-noNLS-His was made using pPJ352 as template. hPanK2(82-570-noNLS)-mCherry and hPanK2(82-570-noNES)-mCherry were made using pAA283 as template. (DOCX) [file pone.0049509.s009.docx]

| **Table S5.** | | | | | |  |
| --- | --- | --- | --- | --- | --- | --- |
| **Name** | **Plasmid** | | **Primer Name** | | **Sequence (5’→3’)** |  |
|  | | | hPanK1a-noNLSa | | AGCATGACTCCCCGGCCGCGGCATGCGCGCTGCGGAGGAGGATGG |  |
|  | | | hPanK1a-noNLSa-rev | | CCATCCTCCTCCGCAGCGCGCATGCCGCGGCCGGGGAGTCATGCT |  |
|  | | | hPanK1anoNLSab | | CGCGGCATGCGCGCTGGCGGCGGCGATGGACTCGGGGAGA |  |
|  | | | hPanK1anoNLSab-rev | | TCTCCCCGAGTCCATCGCCGCCGCCAGCGCGCATGCCGCG |  |
| hPanK1α-noNLS | pAA362 | | hPanK1a_noNLS | | GCGGCGATGGACTCGGGGGCAGCGAACGCGCCGCCATTCCCATGGTT |  |
|  | | | hPanK1a_noNLS-rev | | AACCATGGGAATGGCGGCGCGTTCGCTGCCCCCGAGTCCATCGCCGC |  |
|  | | | mPanK1a_noNLS1 | | GGGATGAGGGGGGCGGCGGCTCCCCGGCC |  |
|  | | | mPanK1_noNLS1-rev | | GGCCGGGGAGCCGCCGCCCCCCTCATCCC |  |
|  | | | mPanK1anoNLS2a | | CCAGCATGATTCTCCGGCCGCGGCATGCGCGCTGCGGAGGAGGATGGAC |  |
|  | | | mPanK1anoNLS2a-rev | | GTCCATCCTCCTCCGCAGCGCGCATGCCGCGGCCGGAGAATCATGCTGG |  |
|  | | | mPanK1anoNLS2ab | | CGCGGCATGCGCGCTGGCGGCGGCGATGGACTCCGGGAGG |  |
|  | | | | mPanK1anoNLS2ab-rev | CCTCCCGGAGTCCATCGCCGCCGCCAGCGCGCATGCCGCG | |
|  | | | | mPanK1noNLS2-f | GGCGATGGACTCCGGGGCGGCGAACGCGCCGCCATTCCCGTGG | |
| mPanK1noNLS1/2 | | pAA363 | | mPanK1a_noNLS2-rev | CCACGGGAATGGCGGCGCGTTCGCCGCCCCGGAGTCCATCGCC | |
|  | | | | hPanK2-NLS1-f | GGCTTCCTGCGGCTTGGGCCAACGGAGCAGGCGGCCGGC | |
|  | | | | hPanK2-NLS1-rev | GCCGGCCGCCTGCTCCGTTGGCCCAAGCCGCAGGAAGCC | |
|  | | | | hPanK2-SDM-NLSb-f | GAGCAGGCGGCGCGCCGGCGGCGGCCCTCTGCTCTG | |
|  | | | | hPanK2-SDM-NLSb-rev | CAGAGCAGAGGGCCGCCGCCGGCGCGCCGCCTGCTC | |
| hPanK2(82-570-noNLS) | | pAA364 | | hPanK2-NES1-f | GGACGTGCACCTCGAGGCGAAGGACGCGACTGCGTGTGGACGCAAAGGC | |
|  | | | | hPanK2-NES1-rev | GCCTTTGCGTCCACACGCAGTCGCGTCCTTCGCCTCGAGGTGCACGTCC | |
| hPanK2(82-570-noNES) | | pAA307 | | hPanK2-NES-f | TCGGGACGTGCACGCCGAGGCGAAGGAC | |
|  | | | | hPanK2-NES-rev | GTCCTTCGCCTCGGCGTGCACGTCCCGA | |
|  | | | | | | |
